# Cerebellar Lobules Optimal Stimulation (CLOS): A Computational Pipeline to Optimize Cerebellar Lobule-Specific Electric Field Distribution

**DOI:** 10.3389/fnins.2019.00266

**Published:** 2019-04-12

**Authors:** Zeynab Rezaee, Anirban Dutta

**Affiliations:** Department of Biomedical Engineering, University at Buffalo, Buffalo, NY, United States

**Keywords:** cerebellum, MRI, non-invasive brain stimulation (NIBS), neuromodulation, finite element analysis

## Abstract

**Objective:**

Cerebellar transcranial direct current stimulation (ctDCS) is challenging due to the complexity of the cerebellar structure which is reflected by the well-known variability in ctDCS effects. Therefore, our objective is to present a freely available computational modeling pipeline for cerebellar lobules’ optimal stimulation (CLOS).

**Methods:**

CLOS can optimize lobule-specific electric field distribution following finite element analysis (FEA) using freely available computational modeling pipelines. We modeled published ctDCS montages with 5 cm × 5 cm anode placed 3 cm lateral to inion, and the same sized cathode was placed on the: (1) contralateral supra-orbital area (called Manto montage), and (2) buccinators muscle (called Celnik montage). Also, a published (3) 4×1 HD-ctDCS electrode montage was modeled. We also investigated the effects of the subject-specific head model versus Colin 27 average head model on lobule-specific electric field distribution. Three-way analysis of variance (ANOVA) was used to determine the effects of lobules, montage, and head model on the electric field distribution. The differences in lobule-specific electric field distribution across different freely available computational pipelines were also evaluated using subject-specific head model. We also presented an application of our computational pipeline to optimize a ctDCS electrode montage to deliver peak electric field at the cerebellar lobules VII-IX related to ankle function.

**Results:**

Eta-squared effect size after three-way ANOVA for electric field strength was 0.05 for lobule, 0.00 for montage, 0.04 for the head model, 0.01 for lobule^∗^montage interaction, 0.01 for lobule^∗^ head model interaction, and 0.00 for montage^∗^head model interaction. The electric field strength of both the Celnik and the Manto montages affected the lobules Crus I/II, VIIb, VIII, and IX of the targeted cerebellar hemisphere where Manto montage had a spillover to the contralateral cerebellar hemisphere. The 4×1 HD-ctDCS montage primarily affected the lobules Crus I/II of the targeted cerebellar hemisphere. All three published ctDCS montages were found to be not optimal for ankle function (lobules VII-IX), so we presented a novel HD-ctDCS electrode montage.

**Discussion:**

Our freely available CLOS pipeline can be leveraged to optimize electromagnetic stimulation to target cerebellar lobules related to different cognitive and motor functions.

## Introduction

Transcranial direct current stimulation of the cerebellum (ctDCS) is a painless non-invasive technique where a weak direct current (i.e., up to 2 mA) is delivered through a scalp electrode overlying the cerebellum (van Dun et al., 2016) which is being explored as a viable intervention for patients with neurological conditions (Grimaldi et al., 2016). This is based on the evidence that cerebellar architecture supports the computations required by the feedforward prediction model from animal studies as well as from studies on patients with cerebellar dysfunction (Ebner, 2013). Specifically, Purkinje cell firing has several of the characteristics of a forward internal model (Ebner, 2013) which is the main target of ctDCS (Galea et al., 2009). For example, Galea and colleagues proposed that ctDCS produces polarity specific effects by polarizing the Purkinje cells thereby affecting the activity in the deep cerebellar output nuclei (Galea et al., 2009). Cerebellar role in modulating sensory processing has also been demonstrated (Popa et al., 2013), which can explain the ctDCS effects on distant plasticity in human cortical areas (i.e., the motor cortex) (Grimaldi et al., 2016). Besides the well-recognized role of the cerebellum in motor function, there is also a concurrent role in cognitive function (Koziol et al., 2014). Most recent works show that cerebellar lobules IV, V, VI, and only a part of VIII is related to motor functions (van Dun et al., 2018) while lobules VI, VII, VIIIa, Crus I and Crus II (Stoodley et al., 2012; Hartzell et al., 2016; Küper et al., 2016; Koppelmans et al., 2017; van Dun et al., 2018) are involved in non-motor functions. Also, Crus I and II have been shown to have no anatomical connections to motor cortex but show projections to the prefrontal cortex (Buckner et al., 2011). Therefore, as we explore ctDCS to affect motor control, cognition, learning and emotions (Ferrucci and Priori, 2014), computation of lobule-specific electric field distribution based on subject-specific head model is necessary for rational dosage considerations (Buckner et al., 2011; Mottolese et al., 2013) e.g., in cerebellar motor syndrome or cognitive performance (Stoodley and Schmahmann, 2009).

Rational dosing of ctDCS needs to account for the very high concentration of neurons with highly organized distribution in the cerebellar cortex. Here, modulation of the activity in the cerebellar neurons with the electric field is the goal (Ferrucci et al., 2015) but is very challenging due to the extreme folding of the cerebellar cortex. Therefore, ctDCS efficacy appears to be limited at present (Ferrucci et al., 2016). It is postulated that the efficacy can be improved significantly by optimizing the ctDCS electrode montage to align the electric field $\overset{\to }{E}$ parallel to the somatodendritic axis (usually radial to gray matter surface for Purkinje cells) that can modulate synaptic efficacy consistent with somatic polarization, with depolarization facilitating synaptic efficacy (Bikson, 2016). Such optimization will require determination of the lobule-specific electric field distribution, $\overset{\to }{E}$, concerning the cerebellar surface to optimize either radial (normal) or tangential components, as necessary. Furthermore, a systematic investigation of subject-specific lobule-specific electric field distribution based on a cerebellar atlas is necessary to investigate the effects of radial (normal) or tangential components of electric field on behavioral and neurophysiological test outcomes. Here, it is critical that the ctDCS electric field is limited to the cerebellar lobules under investigation without spillover to non-targeted regions. However, lobule-specific analysis of subject-specific electric field distribution during ctDCS was not found in the literature (Parazzini et al., 2014; Priori et al., 2014;Fiocchi et al., 2016).

Therefore, the main objective of this technology report is to present a freely available computational pipeline that allows visualization of the lobule-specific electric field distribution during ctDCS. Furthermore, we present an application where the pipeline can be used for the optimization of the lobule-specific electric field distribution which is important to specifically target the architecture of the cerebellar cortex (Stoodley and Schmahmann, 2009). Here, the earliest and the most studied mechanism based on the architecture of the cerebellar cortex is Marr-Albus-Ito hypothesis that assigns specific functions to the climbing fiber-Purkinje cell and the mossy fiber-granule cell-parallel fiber-Purkinje cell circuits (Popa et al., 2016). So, the relative magnitude of the electric field $\overset{\to }{E}$ needs to be quantified in the subject-specific head model (Rahman et al., 2013; Saturnino et al., 2018) to investigate the effects on the climbing fiber-Purkinje cell during ctDCS (Summers et al., 2018). Here, the challenges with lobule-specific targeting of ctDCS include high conductivity of the cerebrospinal fluid (CSF) and extreme folding of the cerebellar cortex (Fiocchi et al., 2017). The goal is an optimal electrode placement, e.g., with more focal high-definition (HD) ctDCS montages (Fiocchi et al., 2017), that can deliver the electric field toward deeper targets by taking advantage of the high conductivity of the CSF and the interhemispheric fissure. Moreover, computational modeling (Parazzini et al., 2014; Priori et al., 2014; Fiocchi et al., 2016) of lobule-specific electric field distribution is important to address the inter-subject variability in the ctDCS effects that is necessary to address for clinical translation (Ferrucci et al., 2016). This is also crucial since ctDCS effects were recently said to be mediated by mechanisms other than cerebellar excitability changes (Grimaldi et al., 2016) where non-focal electric field with two-electrode montages was said to affect brain areas other than cerebellum.

In this technology report, we present a cerebellar lobule’s optimal stimulation (CLOS) pipeline that creates a subject-specific head model based on magnetic resonance imaging (MRI) and then computes the electric field distribution in the cerebellar lobules. Our main contribution is in providing an approach for the isolation of the cerebellum and its lobules based on Spatially Unbiased Infratentorial Template for the Cerebellum (SUIT) atlas (Diedrichsen et al., 2009) that allowed us to investigate the lobule-specific electric fields following finite element analysis (FEA) using different freely available computational pipelines including SimNIBS (Saturnino et al., 2018) and ROAST (Huang et al., 2017). We have adapted the SUIT isolation and activation visualization scripts, which are commonly used to analyze functional MRI activation maps, to analyze the lobule-specific electric fields. Our SUIT–based approach to determine cerebellar lobule-specific electric field distribution can be applied to FEA results for transcranial magnetic stimulation (TMS) too. Here, we found it important to study the effects of subject-specific head model versus Colin 27 average head model on lobule-specific electric field distribution across different freely available computational (FEA) modeling pipelines. To show that visualization of cerebellar lobule-specific electric field distribution can provide further insights, we applied our pipeline to analyze previously published (Abadi and Dutta, 2017) healthy human experimental results during visuomotor learning of myoelectric visual pursuit. During our analysis, we found that the published ctDCS montages used in the study (Abadi and Dutta, 2017) were not optimal for the ankle motor task. For example, posterior and inferior cerebellum (i.e., lobules VI-VIII) is mainly susceptible to the available ctDCS montages (Grimaldi et al., 2016) which may be the reason why recent studies failed to demonstrate a significant association of motor performance and changes in neurophysiological measures after ctDCS (Summers et al., 2018). Therefore, we applied our CLOS pipeline to optimize multi-electrode ctDCS montage to target the cerebellar lobules shown related to ankle functions (Buckner et al., 2011; van Dun et al., 2018). Here, it is important to investigate the effects of the selection of the freely available computational (FEA) modeling pipeline on the lobule-specific electric field distribution across ctDCS montages which is presented in this technology report.

## Materials and Methods

We developed the cerebellar lobule’s optimal stimulation (CLOS) pipeline using freely available software packages that are easily accessible worldwide to facilitate clinical translation of tDCS. Using our CLOS pipeline, we investigated two common ctDCS montages (Grimaldi et al., 2014, 2016) with the anode placed over the right cerebellum, and (1) the cathode placed over the right buccinator muscle – called Celnik montage henceforth, (2) the cathode placed on the contralateral supraorbital area – called Manto montage henceforth. We also investigated a recently published 4 × 1 high-definition (HD) ctDCS montage (Doppelmayr et al., 2016). Our computational pipeline leveraged SUIT, which is one of the automated algorithms developed explicitly for cerebellum segmentation (Diedrichsen, 2006), and is a freely available SPM [Statistical Parametric Mapping (SPM - Statistical Parametric Mapping)] toolbox for functional MRI data analysis. In this toolbox, a probabilistic atlas of the cerebellar nuclei, a cerebellar cortical parcellation atlas in MNI (Montreal Neurological Institute) space, and SUIT template are available. Therefore, we used SUIT SPM toolbox for isolation of cerebellar lobules where SUIT provided an improved and fine-grained exploration, registration and anatomical detail of the cerebellum for structural and electric field images. Since our SUIT–based approach can be applied to FEA results from different freely available FEA software so we compared the lobule-specific electric field results between the freely available SimNIBS pipeline (Opitz et al., 2015) and the Realistic volumetric-Approach to Simulate Transcranial Electric Stimulation (ROAST) pipeline (Huang et al., 2017) using subject-specific head model.

### CLOS Pipeline

#### MRI Data Acquisition and Subject-Specific Head Model Creation

The first step in creating an anatomically accurate subject-specific head model is the segmentation of structural magnetic resonance images (MRI). The individual head model was constructed using MR images taken from a healthy volunteer in accordance with the Declaration of Helsinki - a statement of ethical principles for medical research involving humans. For research participation as well as for the publication of this case report including participant’s identifiable information, written informed consent was obtained from the subject at the University at Buffalo. The subject did not have any history of neurological or psychiatric diseases. Images were taken from 3 Tesla Magnetic Resonance Imaging (MRI) system (Toshiba Vantage) at the University at Buffalo Clinical and Translational Science Institute using a sixteen multichannel receiver head coil. Two T1-weighted images (with and without fat suppression) were acquired for the subject (Windhoff et al., 2013). MR sequence consisted of the following parameters: MPRAGE, 192 slices, matrix size = 256 × 256, Flip/Flop angle = 8/0, TR/TE = 6.2/3.2. Also, two T2-weighted images (with and without fat suppression) were acquired for the subject with the sequence of 30 slices, matrix size of 256 × 256, flip/flop angle of 110/150 degree, and TR/TE = 11990/108. From these four MR images, a tetrahedral volume mesh of the head was created using “mri2mesh” script which is provided in the SimNIBS package (Windhoff et al., 2013). The “mri2mesh” is based on four open source software; FreeSurfer^1^, FSL^2^, Meshfix^3^, and Gmsh^4^. This script integrates all these software into a single pipeline for mesh generation from MR images (Windhoff et al., 2013). After segmentation using FSL and FreeSurfer, five tissues were modeled by the volume mesh; Skin, Skull, Cerebrospinal Fluid, Gray Matter, and White Matter. Different brain tissues for the volume mesh components were modeled as different volume conductors in SimNIBS with their specific conductivity (Windhoff et al., 2013), as shown in Table 1. We also used Colin27 average brain (Holmes et al., 1998), which is the stereotaxic average of 27 T1-weighted MRI scans of the same individual, to create another head model (Guhathakurta and Dutta, 2016) for comparison.

**Table 1:** Electrical conductivity.

Component	Electrical conductivity (S m ^-1^)
Scalp	0.465
Skull	0.010
CSF	1.654
Gray matter	0.276
White matter	0.126


#### Published Electrode Montages for ctDCS

In order to investigate lobule-specific electric field distribution from published ctDCS montages (Galea et al., 2009; Grimaldi and Manto, 2013; Doppelmayr et al., 2016), electrode positions were defined as follows:

(1) Celnik montage (Galea et al., 2009): 5 cm × 5 cm anode was placed over the right cerebellum, 1 cm below and 3 cm lateral to the inion (Iz, 10/10 EEG system), and the 5cm × 5cm cathode was placed over the right buccinator muscle for anodal ctDCS with 2 mA direct current.
(2) Manto montage (Grimaldi and Manto, 2013): 5 cm × 5 cm anode was placed over the right cerebellum, 1 cm below and 3 cm lateral to the inion (Iz, 10/10 EEG system), and the 5 cm × 5 cm cathode was placed on the contralateral supraorbital area (FP2, 10/10 EEG system) for anodal ctDCS with 2 mA direct current.
(3) HD-ctDCS 4×1 montage (Doppelmayr et al., 2016): 3.14 cm^2^ anode was placed above the cerebellum 10% below Oz (10/10 EEG system) in the midline, and four 3.14 cm^2^ cathodes were placed at Oz, O2, P8, and PO8 (10/10 EEG system) for anodal ctDCS with 1 mA direct current.

We investigated the lobule-specific electric field of anodal ctDCS due to the three electrode montages given above using the subject-specific head model as well as the Colin27 head model (Holmes et al., 1998; Guhathakurta and Dutta, 2016).

#### Finite Element Analysis of ctDCS Using SimNIBS

**FIGURE 1:**
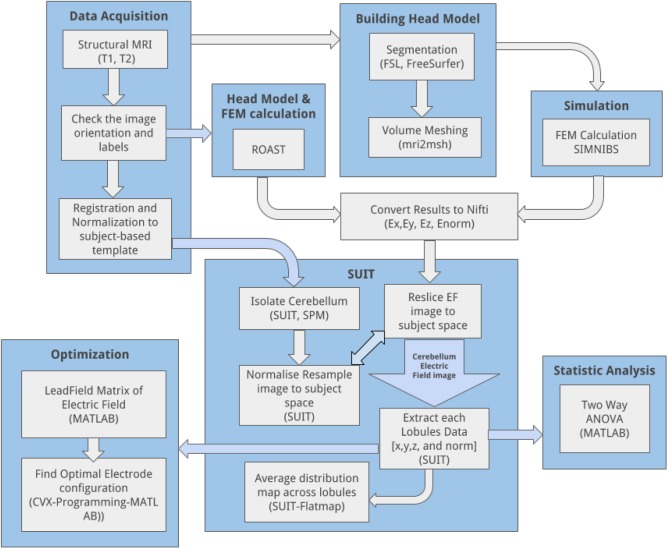
CLOS pipeline: overall workflow to visualize and optimize the electric field distribution across cerebellar lobules during cerebellar transcranial direct current stimulation.

**FIGURE 2:**
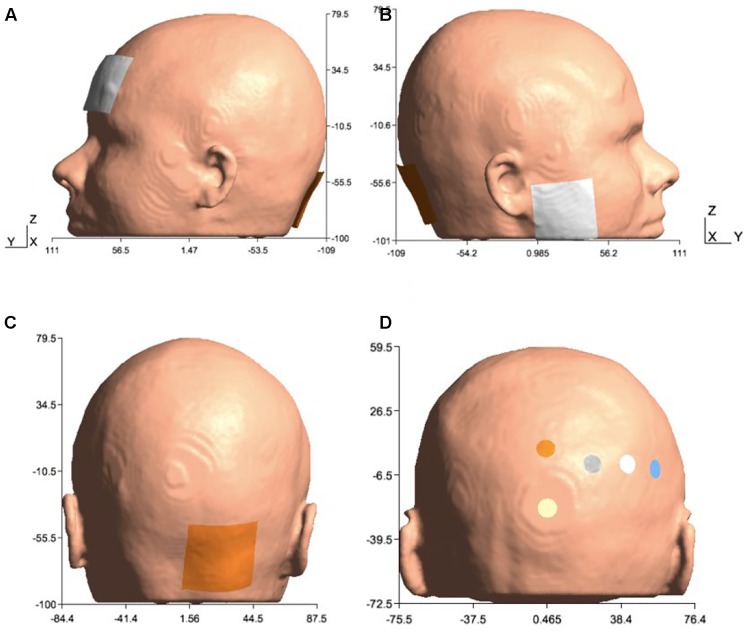
Electrode configurations: **(A)** Manto montage: anode was placed over the right cerebellum, 1 cm below and 3 cm lateral to the inion (Iz, 10/10 EEG system), and the cathode was placed on the contralateral supraorbital area (FP2, 10/10 EEG system), **(B)** Celnik montage: anode was placed over the right cerebellum, 1 cm below and 3 cm lateral to the inion (Iz, 10/10 EEG system) and cathode was placed over the right buccinator muscle, **(C)** Anode was placed over the right cerebellum, 1 cm below and 3 cm lateral to the inion (Iz, 10/10 EEG system) for Manto and Celnik Montages, and **(D)** 4×1 HD-tDCS montage: anode was placed above the cerebellum 10% below Oz (10/10 EEG system) in the midline, and four cathodes were placed at Oz, O2, P8, and PO8 (10/10 EEG system).

Finite element method was used to solve the quasistatic approximation for Maxwell’s equation, ∇⋅ (σ∇Φ) = 0 in Ω [called the Laplace equation (Griffiths, 2017)], where *Φ*is a potential and σ is the conductivity tensor in the volume conductor Ω. The solution to the Laplace equation is unique if the electric field (or, equivalently, the current density) is specified at all the locations. The applied ctDCS current density $\overset{\to }{J_{e}}$ at the electrodes is normal (n^) to the boundary surface Γso (σ∇Φ⋅n^=Je→) at the electrodes while (σ∇Φ⋅n^=0) otherwise on Γ – a mixed boundary condition. Here, finite element analysis (FEA) was conducted on the subject-specific head model as well as the Colin27 average head model (Holmes et al., 1998; Guhathakurta and Dutta, 2016) to estimate the ctDCS induced electric field in the brain tissues. The anodal ctDCS was delivered using two 5 cm × 5 cm electrodes and a direct current of 2 mA. In all the simulations, the voxel size was 1 mm^3^. The anode and the cathode injected the specified amount of current (source) in the volume conductor, i.e., the head model. The electrodes were modeled as a saline-soaked sponge placed at a given scalp location using 10/10 EEG system (Giacometti et al., 2014). We analyzed the head-model for electric field distribution using the SimNIBS pipeline (Windhoff et al., 2013). Following SimNIBS FEA, we used SUIT to isolate the cerebellum in SPM^5^ package in Matlab (The Mathworks Inc., United States). Subject’s T1 images were reoriented into LPI (Neurological) orientation. The isolation map was manually verified in an image viewer (MRIcron). After the isolation, the cerebellum was normalized to the SUIT atlas template using the cropped image and the isolation map. A non-linear deformation map to the SUIT template is the result of the normalization step. After the normalization, we could either resample the image into SUIT space or into the subject space. The latter was chosen for our subject-specific analysis to resample the probabilistic atlas of the cerebellum into the space of the individual subject. We customized msh2nifti script^6^ to save the electric field distribution in the three direction – Ex, Ey, and Ez – from SimNIBS FEA results, as shown by the head model in Figure 2. The msh2nifti script created NIfTI (Neuroimaging Informatics Technology Initiative) images of the electric field distribution that were resliced using the individual mask and deformation matrix found in the previous step – see the workflow in the Figure 1 using the SUIT toolbox to extract the cerebellar regions (or, lobules). The post-processing of the electric field distribution over the tetrahedral volume mesh and its visualization was performed in Gmsh (Geuzaine and Remacle, 2009). The volume of the cerebellar lobules, defined by the SUIT atlas (Diedrichsen, 2006), was used for the extraction of the lobule-specific electric field distribution in Matlab (The Mathworks Inc., United States). To visualize electric field distribution in cerebellar lobules, the flatmap script in SUIT toolbox was used in Matlab (The Mathworks Inc., United States), which provided a flat representation of the cerebellum after volume-based normalization as described by Diedrichsen (2006).

#### Statistical Tests for the Effects of Lobules, Montage, and Head Model on the Lobule-Specific Electric Field Distribution

For group analysis of the lobule-specific electric field distribution, an averaging of the SUIT flatmap across subjects is possible. Here, the electric field distribution across lobules is important to determine the focality of different ctDCS montages. We performed the analysis of variance (ANOVA) of the cerebellar electric field distribution to investigate the factors of interest – lobules (28 from SUIT), montages (Celnik, Manto, HD-ctDCS), head model (Colin27, subject-specific), and their interactions. Also, two-way ANOVA of the cerebellar electric field distribution was conducted with the subject-specific head model to investigate the factors of interest – lobules (28 from SUIT), montages (Celnik, Manto, HD-ctDCS). Post-hoc multiple comparisons of the means (95% significance) were conducted with Bonferroni critical values. In the Generalized Linear Model (GLM), the proportion of the total variability in the dependent variable that is accounted for by the variation in the independent variable was found using the eta-squared effect size measure (Lakens, 2013).

#### Finite Element Analysis of ctDCS Using ROAST

We used another freely available FEA pipeline called Realistic volumetric Approach to Simulate Transcranial Electric Stimulation (ROAST) (Huang et al., 2018) to compare its lobule-specific electric field distribution with that from the SimNIBS pipeline (see Finite Element Analysis of ctDCS Using SimNIBS). We constructed a subject-specific head model using the same T1- and T2-weighted MRI from Section “Finite Element Analysis of ctDCS Using SimNIBS.” The creation of the tetrahedral volume mesh of the head and solving the finite element model were implemented by ROAST. The pipeline is a Matlab script based on three open source software: Statistical Parametric Mapping (SPM) (Penny et al., 2011), Iso2mesh (Fang and Boas, 2009), and getDP (Dular et al., 1998). ROAST provided results for electric field distribution as NIfTI images which were processed in our pipeline to isolate the cerebellum for the analysis of the lobule-specific electric field distribution, as described in Section “Finite Element Analysis of ctDCS Using SimNIBS.”

### Application of the Computational Pipeline to Analyze Experimental Data From a Healthy Human Study

In our published healthy human study (Abadi and Dutta, 2017), 15 healthy volunteers participated in accordance with the Declaration of Helsinki. Ethics approval was obtained at the University Medical Center, Goettingen, Germany. In this study, two-electrode anodal ctDCS montages were investigated for the application of anodal transcranial direct current stimulation over the cerebellar hemisphere during visuomotor learning of myoelectric visual pursuit using the electromyogram (EMG) from ipsilateral gastrocnemius (GAS) muscle. This study was conducted to investigate the effects of 15min of anodal ctDCS (current density = 0.526 A/m^2^; electrode size 5 cm × 5 cm) using Celnik and Manto montage on the response time (RT) and root mean square error (RMSE) during isometric contraction of the dominant GAS for myoelectric visual pursuit, i.e., ‘ballistic EMG control’ (Dutta et al., 2014; see Abadi and Dutta, 2017 for further details). The EMG RT was computed offline as the duration from the instant of visual cursor target cue to the instant when the rectified EMG in a sliding window of 500 ms from the muscle jumped by more than three times of the standard deviation of the resting value. The response accuracy was computed as RMSE between the EMG driven cursor and the cursor target signals during cue presentation. 95% confidence intervals for the parameters were compared for overlap between post-intervention and baseline based on Student’s *t*-distribution.

### Application of the Computational Pipeline to Optimize ctDCS Montage for Cerebellar Lobules Related to Ankle Function

To calculate the optimal ctDCS electrode configuration to target the cerebellar lobules shown related to motor functions (van Dun et al., 2018), especially ankle function (Buckner et al., 2011), we applied convex optimization (Boyd and Vandenberghe, 2004) in MATLAB (The Mathworks Inc., United States). Convex optimization was previously used by Dmochowski et al. (2011) and Guler et al. (2016) for non-cerebellar targets. We leveraged our computational pipeline to first determine the ‘transfer matrix’ (or ‘lead field matrix’) from the electrodes on the scalp to the lobule-specific average electric field. Then, we applied convex optimization to find the electrode montage that minimizes the error from the specified lobule-specific average electric field at cerebellar lobules VII-IX (van Dun et al., 2018).

#### Computation of the ‘Transfer Matrix’ or ‘Lead Field Matrix’ in CLOS Pipeline

Any freely available computational modeling pipeline (see CLOS Pipeline) can be used to solve the quasistatic approximation for Maxwell’s equation with a linear approximation of Ohm’s law in a purely resistive medium Ω. So, we can write in a matrix form $\overset{\to }{E}=LI$ where $\overset{\to }{E}$ is the electric field in the brain generated by stimulation currents, I, applied to an electrode array and L is the ‘transfer matrix’ (or, ‘leadfield matrix’) that quantifies the electric field generated in the brain for a unit current applied to each of the stimulation electrodes (Dmochowski et al., 2011). Here, the problem of choosing an appropriate stimulation currents I for the multi-electrode array to shape the induced electric field is similar to the ‘beamforming’ problem in array signal processing (Dmochowski et al., 2011). Specifically, we formulated a convex optimization problem (Boyd and Vandenberghe, 2004) where we minimized the Euclidean norm of the error between the desired brain activation (i.e., the electric field distribution $\overset{\to }{E}$ at cerebellar lobules VII-IX) and the one generated by the stimulation currents (see CLOS Pipeline), i.e., arg ${\min_{I}||\overset{\to }{E}-LI||}^{2}$. Due to safety and comfort considerations as well as due to restrictions on our tDCS device (StarStim 8, Neuroelectrics), we had constraints on the maximum injected current. Therefore, the ‘leadfield matrix’ or ‘transfer matrix’ is a forward model from the current injection at the scalp electrodes to the electric field in the brain that captured a reduced dimension head model as a Ohmic volume conductor (Dutta and Dutta, 2013). The individual head model in this study was constructed using MR images taken from a healthy volunteer (see MRI Data Acquisition and Subject-Specific Head Model Creation). From these MR images, a tetrahedral volume mesh of the head was created using “headreco” script which is provided in the SimNIBS package (Windhoff et al., 2013; Saturnino et al., 2018). The “headreco” is based on SPM^7^ package in Matlab (The Mathworks Inc., United States). Here, all FEA simulations to compute the ‘leadfield matrix’ or ‘transfer matrix’ used circular electrodes (1 cm diameter) based on the EGI EEG net-based system^8^ with a common cathode at the vertex (Cz) and a direct current of 1 mA. The anode was placed at the EEG locations one by one while the cathode stayed at the vertex (Cz). So, a series of 417 bipolar electrode montages were simulated using our CLOS pipeline (see CLOS Pipeline) and then the ‘leadfield matrix’ (Dutta and Dutta, 2013) was computed for the ‘beamforming’ (Dmochowski et al., 2011) to stimulate the cerebellar lobules VII-IX (Buckner et al., 2011; van Dun et al., 2018).

CLOS pipeline (see CLOS Pipeline) was used to compute the average electric field in the three directions (X, Y, Z) in each of the 28 SUIT lobules (Diedrichsen, 2006) as well as at the non-cerebellar brain. For the non-cerebellar brain, the cerebellum was masked, and the electric field across the rest of the brain was averaged. Then, the ‘transfer matrix’ or the ‘lead field matrix’ was computed for each direction of the electric field by combining 417 FEA simulations where the mapping was from the 417 scalp locations to the 28 SUIT lobules and the non-cerebellar brain. Here, the possible electrode positions were defined for the whole head coverage by combining the high-density 10-05 EEG locations (Oostenveld and Praamstra, 2001) with the EGI net-based system^9^ and extra electrodes from ROAST (Huang et al., 2018). So, we identified a total of 417 scalp locations to consider in our optimization procedure.

#### Computation of the Optimal Electrode Montage Based on ‘lead Field Matrix’

Consider a set of N bipolar electrode montages where the Ohmic relation from the electrode current array, *s* (anode positive current), to the average electric field at a certain lobule, *b*, can be written in a matrix form,

*b = LF ⋅ s* Equation (1)

where

$$b=\left[\begin{array}{c} b_{1}\\ \cdot \\ \cdot \\ \cdot \\ b_{29} \end{array}\right]$$

and


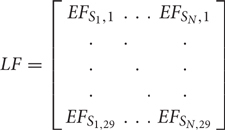


Here, LF is the ‘lead field matrix’ and *b*_1_ to *b*_29_ are the volume-average electric field at the 28 cerebellar lobules along with the non-cerebellum brain (*b*_29_) due to all the N bipolar electrode montages. So, *EF_S_N_,M_* in LF is the volume-average electric field at M*^th^* lobule due to N*^th^* anode delivering 1mA. Linear equation 1 allowed us to write the objective function viz. arg min_x_||LF ⋅ x-b||^2^ that optimized an appropriate electrode current array, *x*, to minimize the L2-norm of the error, (LF. x-b), given a desired electric field distribution, *b*, across 28 cerebellar lobules and the non-cerebellar brain. The following constraints were considered for *x*:

o Total anodal current is equal to the cathodal current;

$$\overset{N}{\underset{n=1}{\Sigma }}x_{n}=0$$

o Total anodal and cathodal current magnitude is below a set threshold of 4mA for safety and comfort (i.e., maximum total anodal or cathodal current is 2 mA);

$$\overset{N}{\underset{n=1}{\Sigma }}|x_{n}|\leq 4$$

The convex optimization problem (Boyd and Vandenberghe, 2004) was solved to get a uniform electric field at the cerebellar lobules related to ankle function (Buckner et al., 2011), a.k.a, lobules VII-IX.

## Results

### Finite Element Analysis of ctDCS Using SimNIBS

**FIGURE 3:**
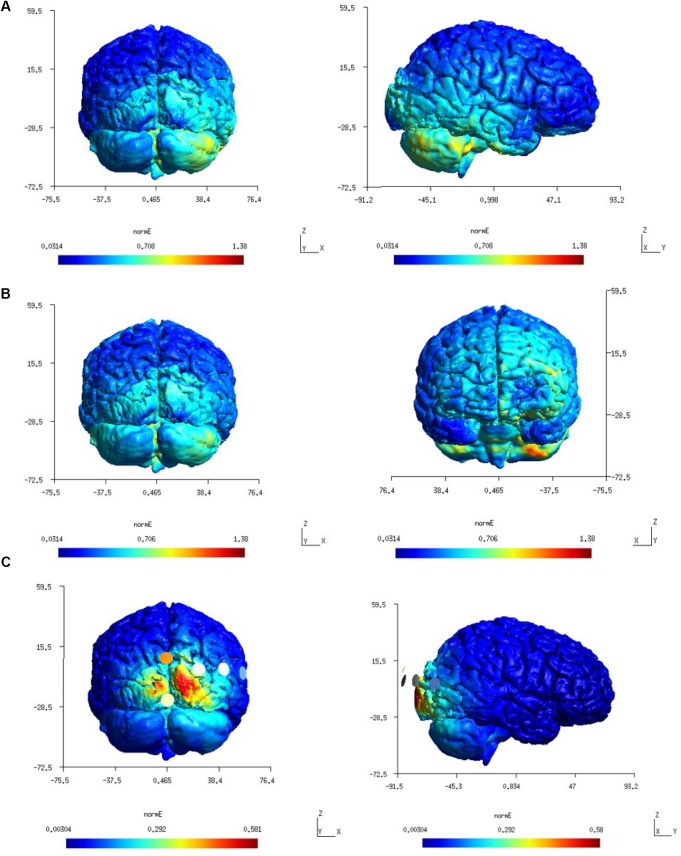
**(A)** first-row panels (color scale: 0.0314–1.38 V/m) – electric field (Enorm) distribution for Celnik montage which was found confined to the right hemisphere. **(B)** second-row panels (color scale: 0.0314–1.38 V/m) – electric field (Enorm) distribution for the Manto montage at the right cerebellum and left prefrontal cortex. **(C)** third-row panels (color scale: 0.00304–0.581 V/m) – electric field (Enorm) distribution for the 4×1 HD-tDCS montage.

Figure 3 shows a higher average electric field strength (magnitude or E*_norm_*) at the targeted right cerebellar hemisphere than the left cerebellar hemisphere since the anode was placed over the right cerebellum (lateral to the inion) in both the Celnik and the Manto montages. FEA using SimNIBS showed that the ctDCS electric field magnitude for both the Celnik and the Manto montages could spread to neighboring structures, e.g., the right temporal lobe for the Celnik montage and the left prefrontal cortex for the Manto montage, as shown in Figure 3A,B. Moreover, ctDCS electric field strength for the 4×1 HD-ctDCS montage proposed by Doppelmayr et al. (2016) can spread to the occipital lobe, as shown in Figure 3C. Here, the central anode for 4×1 HD-ctDCS was placed 10% below the inion, but the cathodes were located at Oz, O2, P8, and PO8 which are partly on the right occipital lobe.

**FIGURE 4:**
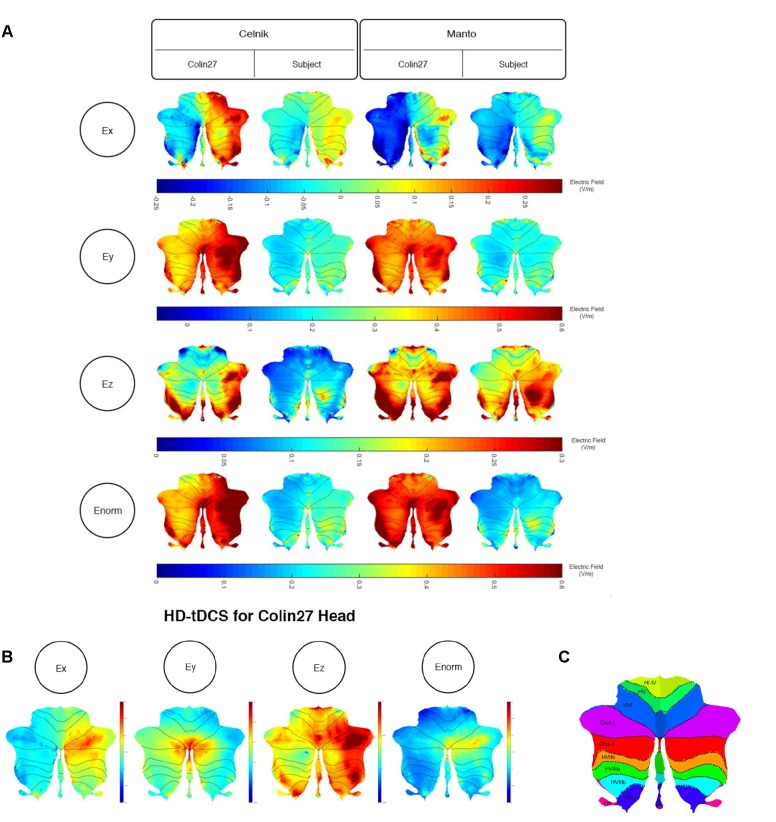
**(A)** Comparison between the SimNIBS outcomes for Celnik and Manto Montages on Colin27 and subject-specific head model. Electric field distribution (Ex, Ey, Ez, and Enorm) of Celnik and Manto montages for Colin27 Head and Subject-specific head model were visualized in SUIT toolbox using flatmap. First row: Color Scale of –0.25–0.3 V/m – Electric field distribution (Ex); second row: Color Scale of –0.05–0.6 V/m – Electric field distribution (Ey); third row: Color Scale of 0–0.3 V/m – Electric field distribution (Ez); fourth row: Color Scale of 0–0.6 V/m – Electric field distribution (Enorm). **(B)** Electric field distribution of 4×1 HD-tDCS montage for Colin27 head model: Ex (color scale: –0.02 to 0.06 V/m), Ey (color scale: 0–0.18 V/m), Ez (color scale: 0–0.5 V/m), and Enorm (color scale: 0–0.15 V/m). **(C)** SUIT lobules (Diedrichsen et al., 2009).

We further analyzed the SimNIBS FEA results using our SUIT-based computational pipeline to compute lobule-specific electric field distribution. The SUIT flat map results for E_x_, E_y_, E_z_, and E_norm_ are shown in Figure 4A (and the volume-averaged quantitative values are presented in the Supplementary Tables 1–3 of the Supplementary Material). E_y_, which is approximately normal to the scalp surface at the anode, has the highest strength (maximum 0.6 V/m) while E_x_ and E_z_ have comparable strength (maximum 0.3 V/m). The SUIT flat map results for E*_norm_* (in Figure 4A) showed that the Celnik and Manto montages primarily affected the Crus I/II, VIIb, VIII, and IX of the targeted right cerebellar hemisphere. However, Manto montage had a more spillover to the contralateral left cerebellar hemisphere than the Celnik montage (see Supplementary Tables 1–3 of the Supplementary Material). Also, When compared with the Colin27 head model, our subject-specific head model resulted in an overall lower magnitude for the electric field distribution – an effect of the head model shown in Figure 4A. Here, Figure 4A also shows the subject-specific differences in the lobule-specific electric field distribution when compared to the Colin27 head model. This demonstrated the importance of subject-specific optimization of the electrode montage. Figure 4B showed that the 4×1 HD-ctDCS montage led to more focal electric field strength (E_norm_) at the Crus I, Crus II of the targeted right cerebellum (see also Supplementary Table 3 of the Supplementary Material), however, the magnitude (E_norm_) was much lower due to a smaller (1mA) direct current at the anode. Although the current intensity at the anode was lower for 4×1 HD-ctDCS, the current density at the electrode-skin interface was much higher at 0.32 mA/cm^2^ when compared to only 0.08 mA/cm^2^ for the Celnik and the Manto montages.

**FIGURE 5:**
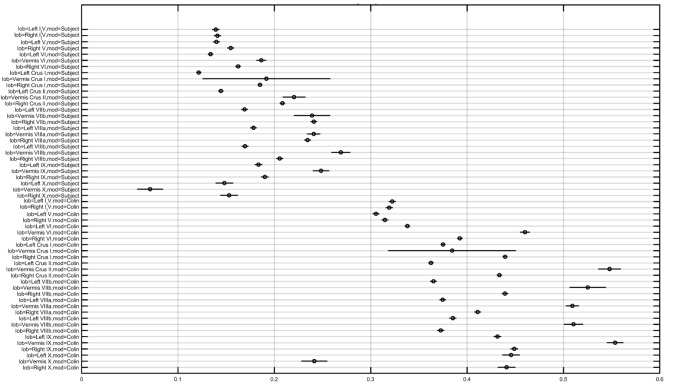
Multiple comparison results for lobule^∗^head model interaction for electric field strength (Enorm is the *X*-axis in the plot in V/m).

In order to investigate the effect of the head model, montage, and lobule on the electric field strength (E_norm_), we computed a three-way analysis of variance (ANOVA). The ANOVA results were evaluated for statistical significance using the eta-squared effect size measure. We found that the eta-squared effect size was 0.05 for lobule, 0.00 for montage, 0.04 for the head model, 0.01 for lobule^∗^montage interaction, 0.01 for lobule^∗^ head model interaction, and 0.00 for montage^∗^head model interaction in case of E_norm_. The lobule^∗^head model interaction for the electric field strength (Enorm) is shown in Figure 5, which shows that the magnitudes are different across head model while the overall electric field (E_norm_) distribution is comparable. If we consider only the Colin27 head model then the two-way ANOVA of the electric field strength (E_norm_) and the post-hoc multiple comparisons of the means (95% significance) with Bonferroni critical values showed that the Celnik and Manto montages primarily affected the lobules Crus I/II, VIIb, VIII, IX of the targeted right cerebellar hemisphere – see Figure 4A, 5 (and the volume-averaged quantitative values are presented in the Supplementary Tables 1, 2 of the Supplementary Material). Post-hoc multiple comparisons of the means (95% significance) of the E_norm_, E_x_, E_y_, and E_z_ are shown in Supplementary Figure 1. Here, the eta-squared effect size measure from two-way ANOVA results was 0.03 for lobule, 0.05 for montage, and 0.02 for interaction in case of E_norm_; 0.38 for lobule, 0.02 for montage, and 0.07 for interaction in case of E_x_; 0.03 for lobule, 0.05 for montage, and 0.02 for interaction in case of E_y_; 0.09 for lobule, 0.04 for montage, and 0.04 for interaction in case of E_z_. Here, the effect sizes are mostly small except for lobule^∗^montage interaction for Ex and lobule for Ez, which were moderate. Manto montage was found to have a spillover to the contralateral cerebellar hemisphere when compared to Celnik montage. Electric field strength for 4×1 HD-ctDCS primarily affected the lobules Crus I, Crus II of the targeted right cerebellar hemisphere – see Figure 4B (also, Supplementary Figure 1A). An interesting finding is the mostly opposite direction of the E_x_ electric field in contralateral (non-targeted hemisphere) cerebellar lobules in the Manto montage when compared to the Celnik montage – see Figure 4A (also, Supplementary Figure 1A and Supplementary Table 3). *Post hoc* multiple comparisons on E_x_, E_y_, E_z_ (Figure 1B–D in the Supplementary Material respectively) also revealed that lobule-specific E_y_ distribution was different for the 4×1 HD-ctDCS montage when compared to Manto and Celnik montages (as shown in Supplementary Figure 1C) where E_y_ for 4×1 HD-ctDCS primarily targeted the vermis region. The average electric field strength (E_norm_) across different lobules for Celnik, Manto, and 4×1 HD-ctDCS montages are listed in Supplementary Tables 1–3, respectively.

### Effects of the Selection of the Freely Available Computational Pipeline on Lobule-Specific Electric Field Distribution Across Different ctDCS Montages

**FIGURE 6:**
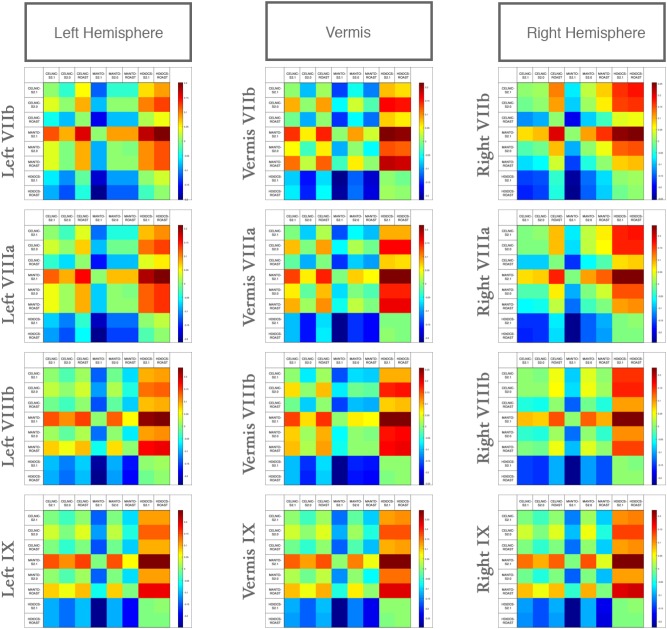
Differences in the lobular electric field strength at VIIb, VIIIa, VIIIb, IX due to different computational pipelines (SimNIBS version 2.1, S2.1; SimNIBS version 2.0, S2.0, and ROAST) for Celnik, Manto, and 4×1 HD-ctDCS montages. Color scale shows the difference across different computational pipelines and ctDCS montages.

We compared the effect of the selection of freely available computational pipeline – SimNIBS (versions 2.0 and 2.1) and ROAST pipelines – on lobule-specific electric field strength across different ctDCS montages. Since VIIb, VIII, IX are related to the lower-limb movements (Buckner et al., 2011; Mottolese et al., 2013) so Figure 6 shows that the electric field strength in those lobules can be affected by choice of the computational pipeline to compute the subject-specific electric field distribution. The lobular electric field strength for the same ctDCS montage can show a different up to ± 0.25 (shown by the color scale) for the lobules VIIb, VIII, IX. Here, SimNIBS version 2.0 (S2.0) took much more time (∼8–10 h) when compared to SimNIBS version 2.1 (S2.1) and ROAST (15–30 min) that leveraged the volumetric segmentation from SPM (Huang et al., 2017). Huang et al. (2017) have already shown a high deviation of SimNIBS version 2.0 generated electric field when compared to SPM-generated result in ROAST which was also found in the lobule-specific electric field distribution for the relevant lobules VIIb, VIII, IX shown in Figure 6 (also see Supplementary Figures 2, 3). Huang et al. (2017) postulated that this difference comes mainly from the two different segmentation approaches, and described the volumetric approach of segmentation in the ROAST pipeline being more realistic of the anatomy when compared to the surface-based segmentation in SimNIBS version 2.0. We found that the components with intersecting surfaces (e.g., gray matter and cerebellum) were better captured by ROAST and SimNIBS version 2.1which led to a better estimate of the bilateral electric field for the Manto montage (see Supplementary Figures 2, 3). These indicate a genuine difference in these two categories of modeling methods where ROAST and SimNIBS version 2.1 performed better (also highlighted by Huang et al., 2017). Importantly, the limitation with SimNIBS version 2.0 is the difficulty in capturing the fine details of the cerebellum which is important for computing the lobule-specific electric field distribution despite the complexity of the cerebellar structure.

### Application of the Computational Pipeline to Analyze Experimental Data From Healthy Human Study and Optimization of the ctDCS Montage for Ankle Function

**FIGURE 7:**
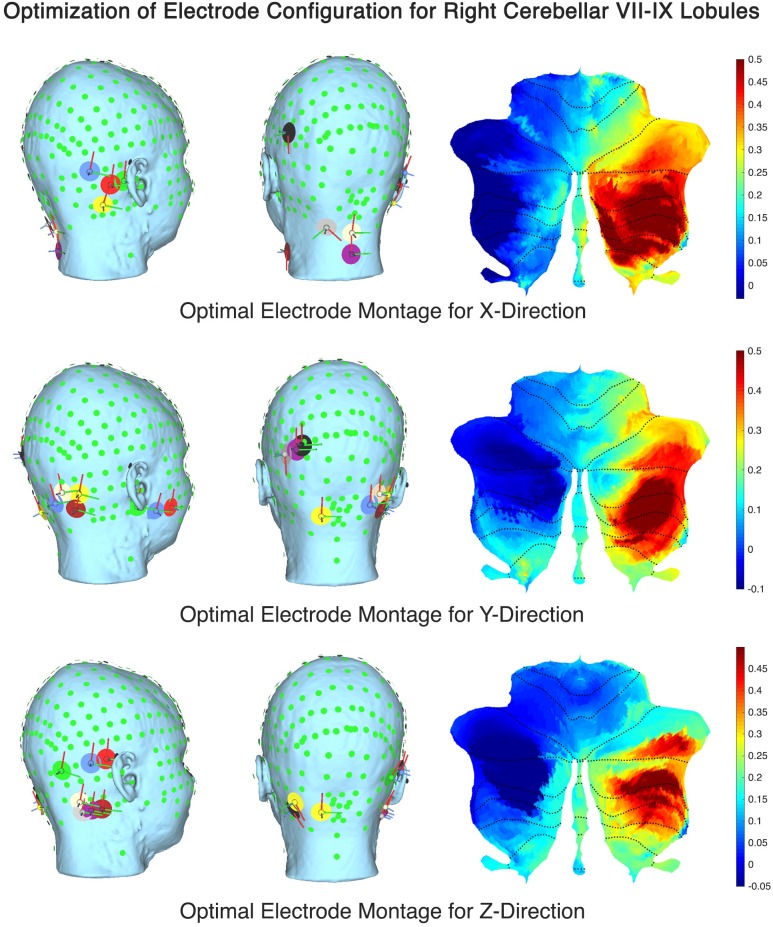
Optimal ctDCS electrode placements for targeting cerebellar lobules VII-IX with electric field (V/m in color scale) in X, Y, and Z directions.

The computational SUIT-based analysis presented in this technology report was used to investigate healthy human anodal ctDCS results during VMT performance (Foerster et al., 2015). Our prior experimental results (Abadi and Dutta, 2017) showed that Manto montage resulted in a statistically significant (*p* < 0.05) decrease in RT post-intervention than baseline when compared to the Celnik montage while Celnik montage resulted in a statistically significant (*p* < 0.05) decrease in RMSE post-intervention than baseline when compared to the Manto montage. Also, ctDCS using Celnik montage has shown to affect the adaptation rate of spatial but not the temporal elements of walking (Jayaram et al., 2012) which is postulated to be related to electric field effects on different cerebellar regions, e.g., vermis (for spatial) versus adjacent hemispheres (for temporal elements) (Jahn et al., 2004). Indeed, we found in our analysis using CLOS pipeline that Celnik montage had a more unilateral effect of the electric field strength (E_norm_) on the cerebellar hemispheres including vermis, as shown in Figure 4. Due to this limitation with published ctDCS montages, we aimed to optimize the ctDCS electrode locations to target ankle function during VMT (Abadi and Dutta, 2017), a.k.a, lobules VII-IX (Buckner et al., 2011). Figure 7 shows the ctDCS electrode placements to target the cerebellar lobules VII-IX with an electric field (V/m in color scale) in X, Y, and Z directions. The flat map shown on the right panel demonstrates that CLOS optimization was successful where the hotspot with the peak electric field targeted the cerebellar lobules VII-IX. Supplementary Table 4 in the Supplementary Material presents the quantitative results of the current intensity at different electrode locations to align the electric field in X, Y, and Z directions to target different lobules (Right VIIb-VIII-IX, VIIb-VII-IX, Right CrusII-VIIb-VIII-IX).

## Discussion

Our freely available CLOS modeling approach to optimize lobule-specific electric field distribution based on subject-specific MRI provided an insight into healthy human anodal ctDCS results during a VMT performance (Foerster et al., 2015; Abadi and Dutta, 2017). The Celnik and Manto montages in the subject-specific head model affected primarily the lobules Crus II, VIIb, VIII, IX of the targeted cerebellar right hemisphere as shown in Figure 4. This was confirmed by the two-way ANOVA of the cerebellar electric field strength (E_norm_) and the post-hoc multiple comparisons of the means (95% significance) with Bonferroni critical values. Specifically, in the E_x_ direction, Celnik montage performed better than Manto montage while in E_z_ direction, Manto montage performed better than Celnik montage (see Figure 4 in the Supplementary Material). Therefore, the E_z_ electric field due to Manto montage is postulated to be responsible for a statistically significant (*p* < 0.05) decrease in RT post-intervention than baseline, i.e., temporal aspects (Jahn et al., 2004), while the E_x_ electric field due to Celnik montage is postulated to affect the spatial aspect of the target pursuit during VMT (Abadi and Dutta, 2017) and resulted in a statistically significant (*p* < 0.05) decrease in RMSE post-intervention than baseline. Figure 4 also shows that the electric field in the mediolateral (X) direction changed direction across hemispheres for the Manto montage, going from a minimum of -0.25 V/m in the left cerebellum to a maximum of 0.3 V/m in the targeted right cerebellum, which is in contrast to that in the Celnik montage that was mostly positive from 0 to 0.3 V/m. Moreover, Figure 4A (also Supplementary Figure 1A) shows that Celnik montage affected the superior posterior cerebellar hemisphere more effectively than the Manto montage. This is important due to the existence of efferent projections from the superior posterior cerebellar hemisphere (specifically, lobule VI) to the foot area of the primary motor cortex (Lu et al., 2007). Therefore, the ctDCS effects on RMSE by Celnik montage may be the result of motor adaptation based on the ctDCS-modulation of the synaptic activity between parallel fiber and the dendritic tree of the Purkinje cells (i.e., the matrix memory) in the efferent pathways while the decrease in RT to unanticipated visual cue by Manto montage may be the result of ctDCS-enhanced responsiveness of the Purkinje cells in the lobules VIIb–IX related to the lower-limb movements (Buckner et al., 2011; Mottolese et al., 2013).

The electric field distribution in X, Y, Z directions for the lobules associated with the lower-limb movements (hemisphere VIIb–IX) (Mottolese et al., 2013) is shown in Supplementary Figure 4. Here, the effect of the selection of the computational pipeline on the lobule-specific electric field distribution can be up to ± 0.25 for the cerebellar lobules VIIb, VIII, IX for different ctDCS montages as shown in Figure 6 (all the lobules are shown in Supplementary Figure 3). The multiple comparison results for lobule^∗^head model interaction in the cerebellar electric field strength is shown in Figure 5. Since ctDCS montage for ankle function (Buckner et al., 2011), a.k.a, lobules VII-IX is important for an insight into healthy human anodal ctDCS results during a VMT performance (Foerster et al., 2015; Abadi and Dutta, 2017) so we optimized the ctDCS montage for lobules VII-IX as shown in Figure 7. This ctDCS montage is relevant for posture and gait which are sensorimotor actions that involve peripheral, spinal, and supraspinal structures (Jahn et al., 2004) related to motor function which is our future work. However, 4×1 HD-ctDCS montage primarily affected the lobules Crus I, Crus II, VIIb of the targeted cerebellar hemisphere that is linked to cognitive impairments (Stoodley and Schmahmann, 2010) with no anatomical connections to motor cortex. Therefore, 4×1 HD-tDCS montage presented by Doppelmayr et al. (2016) may be relevant for cognitive rehabilitation but not for lower limb motor rehabilitation.

Our optimized the ctDCS montage for lobules VII-IX, as shown in Figure 7, can facilitate rehabilitation of impaired standing balance which is a common problem in persons with multiple sclerosis (pwMS) (Bennett and Leavitt, 2018). Here, movement inefficiency and postural control impairment in pwMS may lead to falls and fatigue. Therefore, locomotor rehabilitation can address efficient sensory-motor integration with balance and eye movement exercises (BEEMS) (Hebert et al., 2018) in conjunction with ctDCS of lower limb function. Here, one should take into account the subcortical route besides the cortical route for the lower limb effects of ctDCS (and cerebellar TMS) where an obvious candidate for the subcortical route is the red nucleus (Mottolese et al., 2013) via superior cerebellar peduncle (SCP). Also, newly named endorestiform nucleus (Human Brainstem - 1st Edition) in the inferior cerebellar peduncle (ICP), at the junction between the brain and spinal cord, may be relevant for ctDCS (and cerebellar TMS) since Postural Assessment Scale for Stroke Patients (PASS) test scores could be predicted by the fractional anisotropy of the ICP (discussion with Dr. Jaillard, CHU Grenoble, France) (Abadi and Dutta, 2017). Indeed, this role of other pathways (ICP, SCP) need further investigation since cerebellar TMS using 110 mm double cone coil (Magstim, United Kingdom) with 1A/us current gradient at the right cerebellar cortex (3 cm lateral to the inion) showed the peak electric field strength at the Crus II (Supplementary Figure 5) which has been shown to have no anatomical connections to motor cortex (Buckner et al., 2011). Furthermore, prior work has suggested that cerebellar TMS has sufficient functional resolution to affect nodes of individual networks within the cerebellum which is also shown by our lobule-specific electric field modeling in Figure 5 of the Supplementary Material. Such lobule specific electric field modeling using individual MRI is crucial for cerebellar TMS due to lack of a motor evoked response to find the “hotspot”. NIBS of the cerebellum is postulated to modulate via thalamic connectivity to M1 since the feedback projections to the cerebral cortex from the cerebellum are conveyed from the deep cerebellar nuclei, principally the dentate nucleus, that terminate in the thalamus. Here, Crus II TMS has been shown to alter default network connectivity within the thalamus while midline TMS was found to not alter default network functional connectivity. Therefore, SUIT high-resolution atlas template of the human cerebellum and brainstem is also crucial for systematic testing of cerebellar brain inhibition (CBI) protocols (Fernandez et al., 2018) using cerebellar TMS which is our future work.

One limitation of this technology report is the lack of neurophysiological testing of our optimized ctDCS montage for ankle function. For example, CBI is a physiological parameter of the connectivity strength between the cerebellum and the primary motor cortex (M1) that can be identified using TMS (Fernandez et al., 2018). Moreover, one may need to use *in vivo* intracranial cerebellar recordings in humans for experimental validation (Huang et al., 2017). Another limitation is an uncertain (anisotropic) conductivity profile for the cerebellum that can have a substantial influence on the prediction of optimal ctDCS montage, e.g., (Schmidt et al., 2015). Indeed, an individualized protocol for ctDCS that is verified with neurophysiological testing is necessary to reduce inter-individual variability (Iodice et al., 2017). Therefore, our freely available CLOS pipeline for cerebellum that is easily accessible worldwide is crucial to facilitate the clinical translation of ctDCS. Besides neurophysiological testing, behavioral system analysis using an error clamp design of the VMT (Kha et al., 2018) may further elucidate the behavioral mechanism of ctDCS. For example, when no visual feedback is presented after motor adaptation during ‘error clamp’ trials (Vaswani and Shadmehr, 2013). It has been postulated that motor memories show little decay in the absence of error if the brain is prevented from detecting a change in task conditions (Vaswani and Shadmehr, 2013). Therefore, during ‘error clamp’ trials, Celnik montage should have little effect on both RMSE and RT while Manto montage is postulated to have a significant effect on RT and little effect on RMSE. We have found RT effect of the primary motor cortex (M1) tDCS that changed the input-output function of the pyramidal cells (Lafon et al., 2017) leading to response time improvement post-tDCS when compared to pre-tDCS baseline performance (Kha et al., 2018). Furthermore, a systematic evaluation of more focal electrode montages, such as multi-anode (1 cm radius) tDCS (Otal et al., 2016), may elucidate the specificity of the ctDCS effects as shown computationally possible for lower limb function in Figure 7. Here, multi-anode (1 cm radius) tDCS may reach deeper into the cerebellum while limiting diffusion to neighboring structures (Fiocchi et al., 2017). From the Doppelmayr’s study (Doppelmayr et al., 2016), we expected a more focal electric field distribution in 4×1 HD-ctDCS montage. However, we found diffusion to neighboring structures, e.g., the occipital lobe, as shown in Figure 3B. Therefore, the visual cortex effects of 4×1 HD-ctDCS (Doppelmayr et al., 2016) need to be evaluated using neurophysiological testing in future studies.

Here, we postulate that subject-specific ctDCS electric field orientation within the cerebellar lobules also needs to be optimized based on subject-specific head modeling where our CLOS pipeline can be useful (see Figure 7). The direction of the electric field vector requires investigation using multi-scale modeling (Seo and Jun, 2017) vis-à-vis Purkinje cell, climbing fiber, and parallel fiber orientations at each lobule, which is our future work. This is motivated by the differences in the electric field in the mediolateral (X) direction that may affect the parallel fibers differently between Celnik and Manto montages due to the difference in the E_x_ direction (see Supplementary Figure 4). Also, in this technology report, the top panel of Figure 4 shows that the electric field in the mediolateral (X) direction is all negative for Manto montage when compared to Celnik montage for the targeted right hemisphere which may be relevant. Therefore, it can be postulated for the cerebellar lobules VII-IX related to ankle function based on our prior work that the E_x_ is primarily responsible for the RMSE post-intervention than baseline while the E_z_ is primarily responsible for the RT post-intervention than baseline, i.e., primarily the temporal aspects. Here, optimization using multi-anode (1cm radius) tDCS (Otal et al., 2016) instead of the single anode in 4×1 HD-ctDCS distributed the total current across the anode and provided a more focal targeting in different directions of the electric field vector (see Figure 7). Moreover, the direction of the electric field vector (shown in Figure 7) can be better controlled by current steering using multi-anode (1cm radius) tDCS (Otal et al., 2016), e.g., in aligning the major axis of the electric field gradient with the cerebellar peduncles, which may be relevant for motor neurorehabilitation (Dutta et al., 2014; Abadi and Dutta, 2017).

## Author Contributions

ZR conducted computational modeling under the guidance of AD. All the authors have drafted the work and revised it critically and have approved the final version before submission.

## Conflict of Interest Statement

The authors declare that the research was conducted in the absence of any commercial or financial relationships that could be construed as a potential conflict of interest.

## Footnotes

1 https://surfer.nmr.mgh.harvard.edu/

2 https://fsl.fmrib.ox.ac.uk/fsl/fslwiki

3 https://github.com/MarcoAttene/MeshFix-V2.1

4 http://gmsh.info/

5 http://www.fil.ion.ucl.ac.uk/spm/

6 https://github.com/ncullen93/mesh2nifti

7 http://www.fil.ion.ucl.ac.uk/spm/

8 https://www.egi.com

9 https://www.egi.com
