# Supplementary material for: Cerebellar Lobules Optimal Stimulation (CLOS): A Computational Pipeline to Optimize Cerebellar Lobule-Specific Electric Field Distribution
Source: Front Neurosci. 2019 Apr 12;13:266. doi: 10.3389/fnins.2019.00266 (PMC6473058; doi:10.3389/fnins.2019.00266)
Supplement: Supplementary file 1 [file Data_Sheet_1.pdf]

Supplementary Figure 1

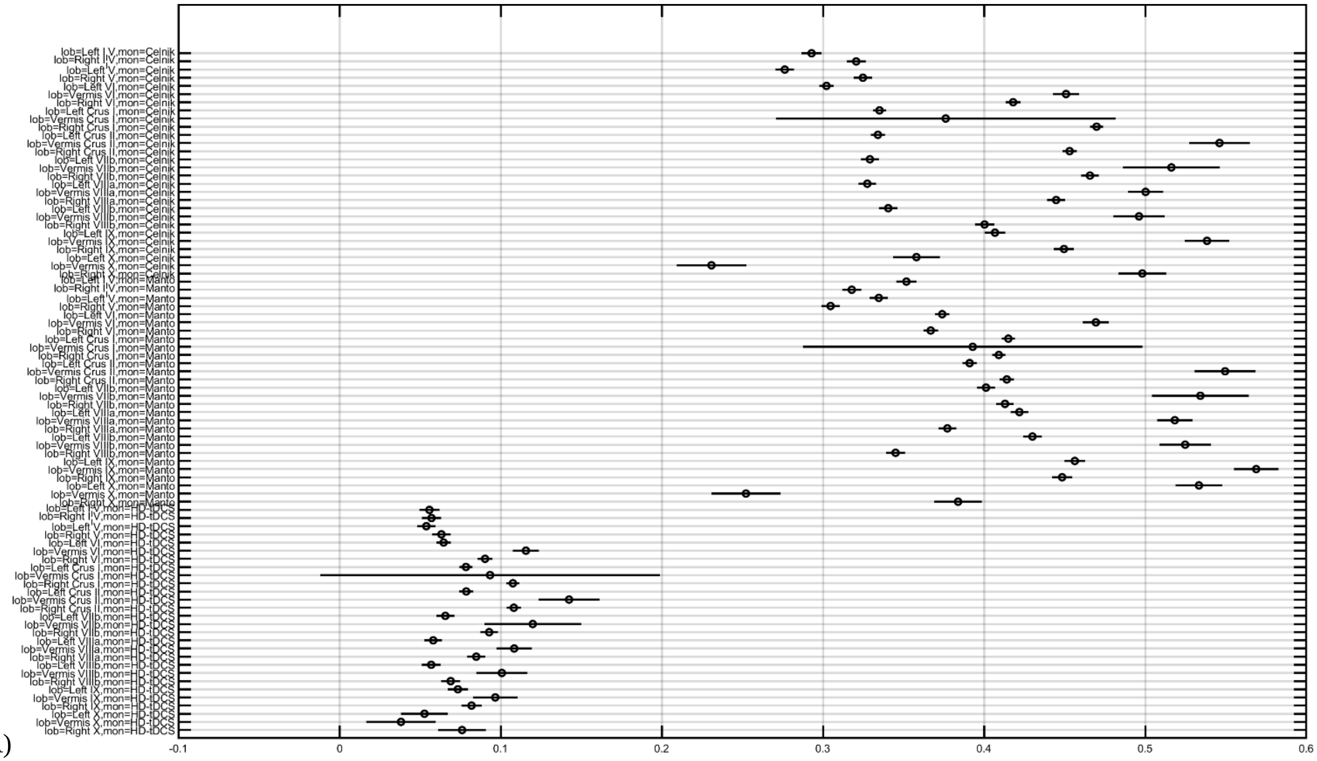

A)

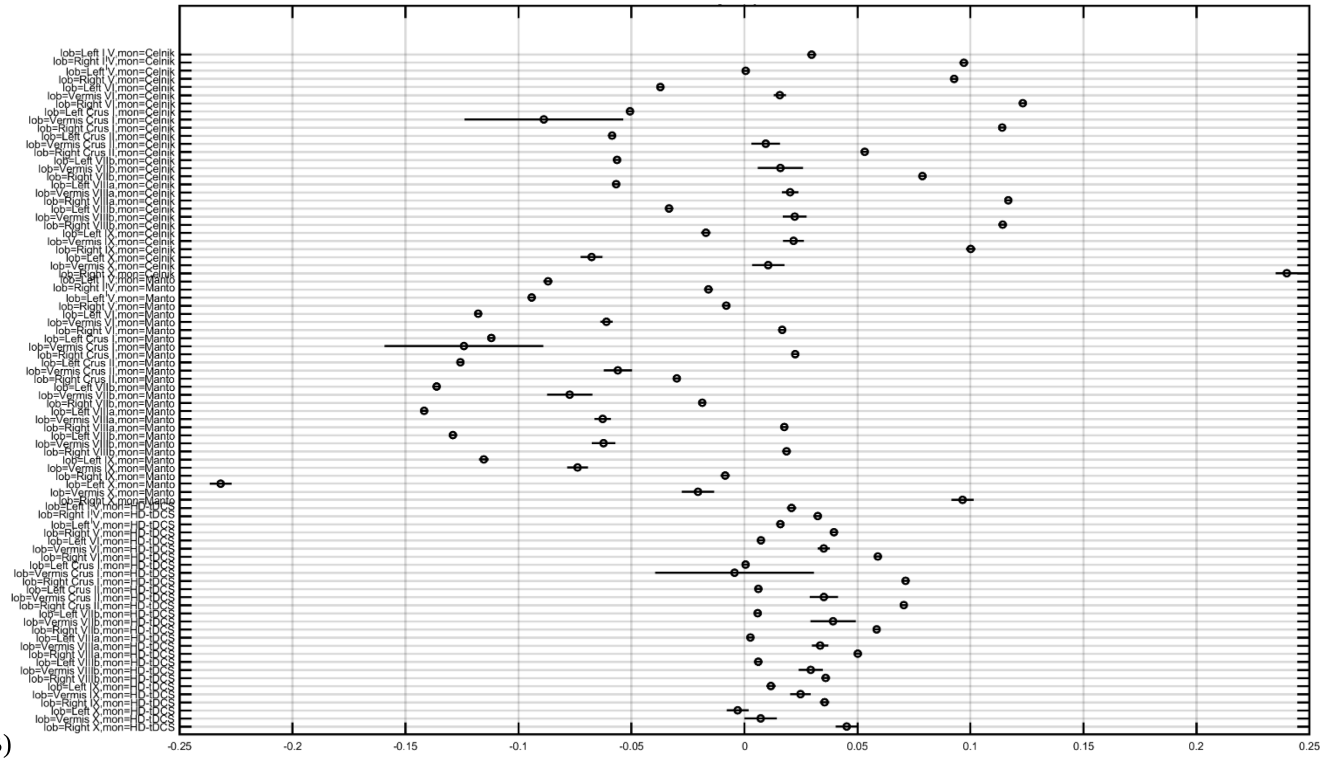

B)

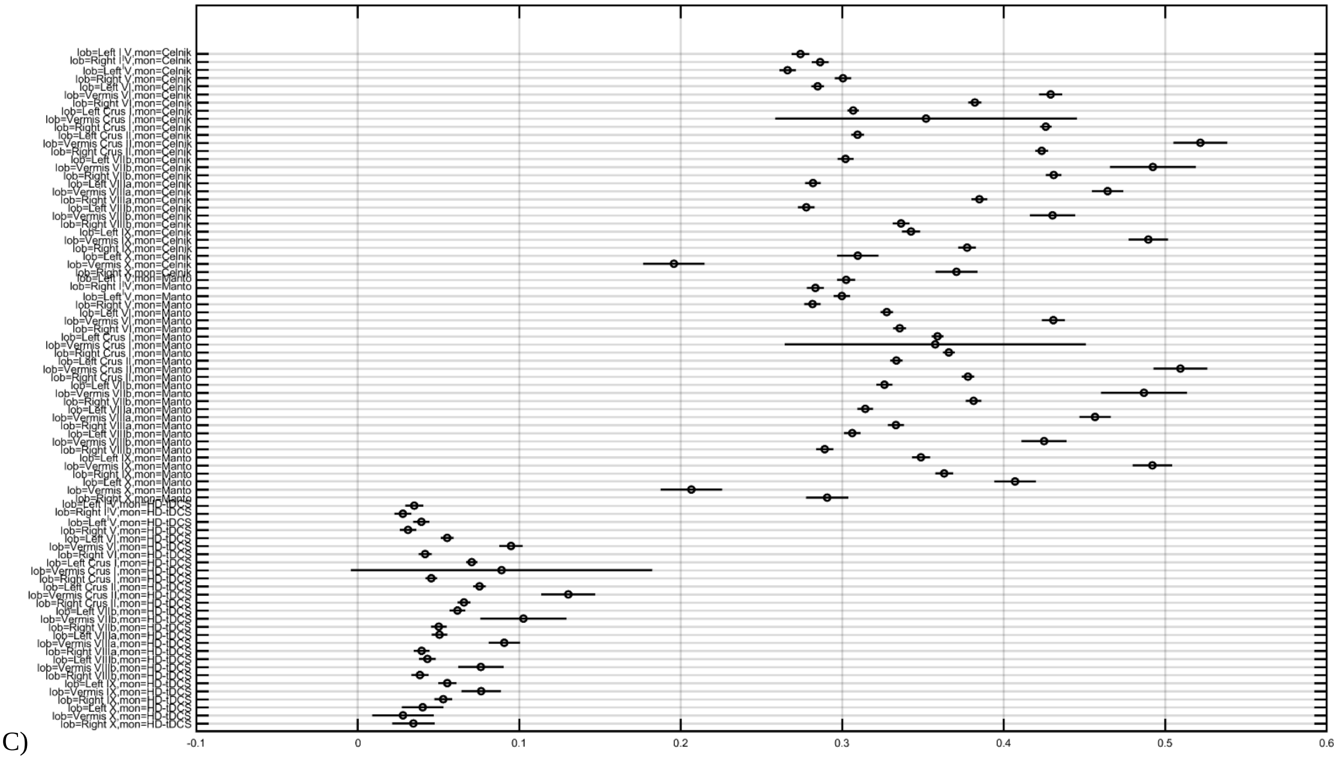

C)

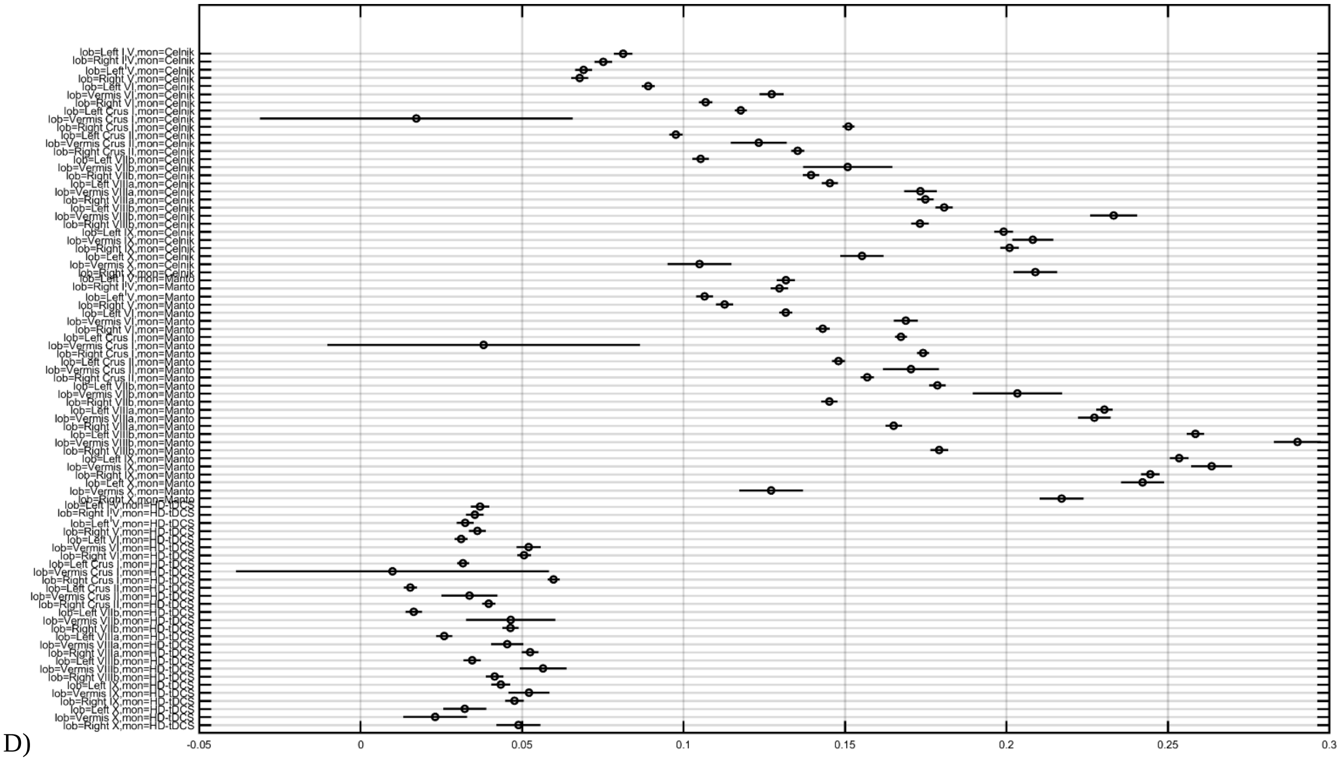

D)

Figure 1. Post-hoc multiple comparison with Bonferroni critical values for Celnik, Manto, and HD-ctDCS montages with Colin27 head model for electric field distribution (x-axis is in V/m) with the horizontal lines showing a 95% confidence interval for the true difference of the means (shown with a circle): A) E_{norm}, B) E_x, C) E_y, D) E_z.

Supplementary Figure 2

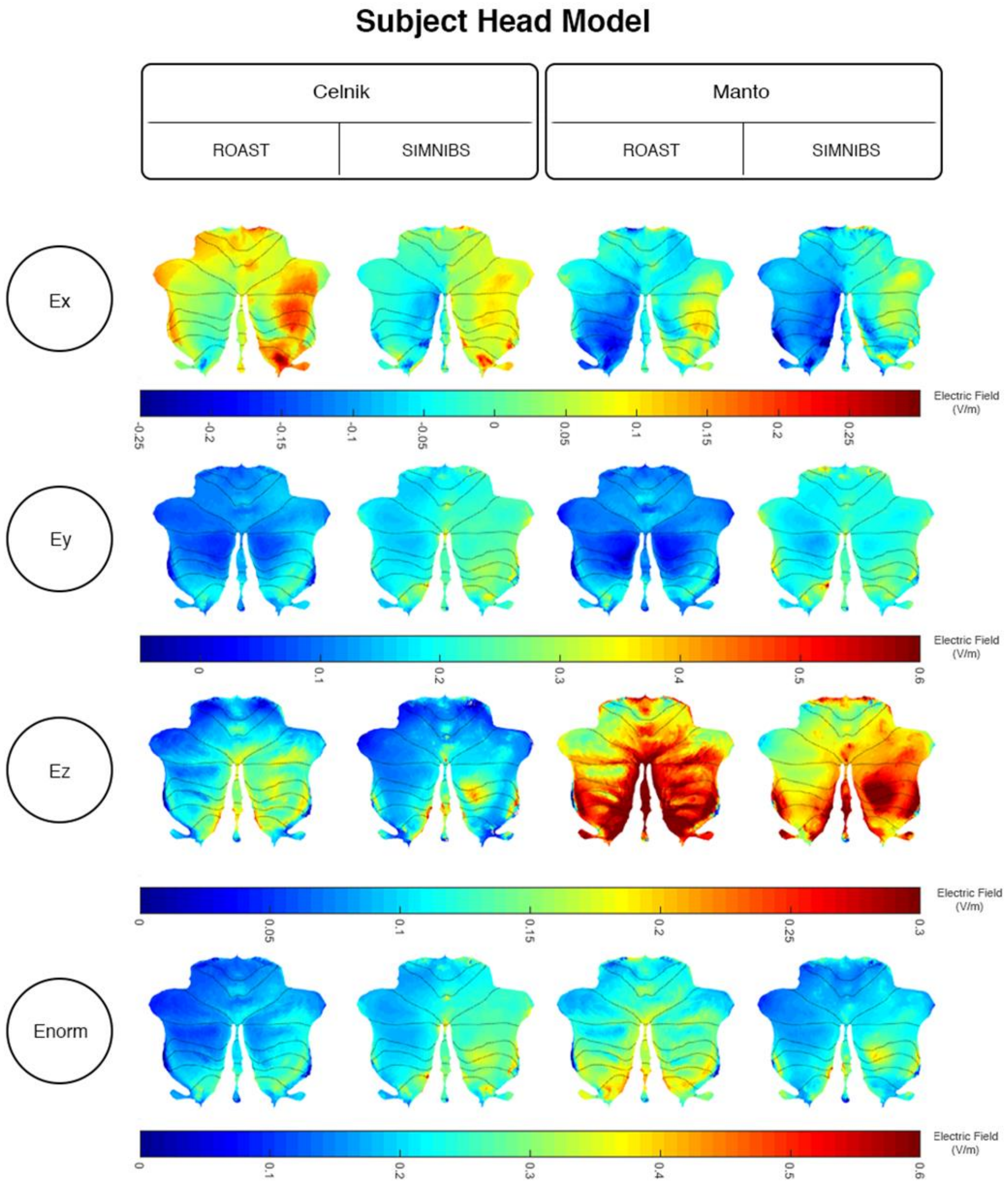

Figure 2. Comparison of the SimNIBS version 2.0 and ROAST outcomes for Celnik and Manto Montages for the subject-specific head model. Electric field distribution (E_x, E_y, E_z, and E_{norm}) of Celnik and Manto montages for Subject-specific head model were visualized in SUIT toolbox using flatmap. First row: Color Scale of –0.25 to 0.3 V/m – Electric field distribution (E_x); second row: Color Scale of -0.05 to 0.6 V/m – Electric field distribution (E_y); third row: Color Scale of 0 to 0.3 V/m – Electric field distribution (E_z); fourth row: Color Scale of 0 to 0.6 V/m – Electric field distribution (E_{norm}).

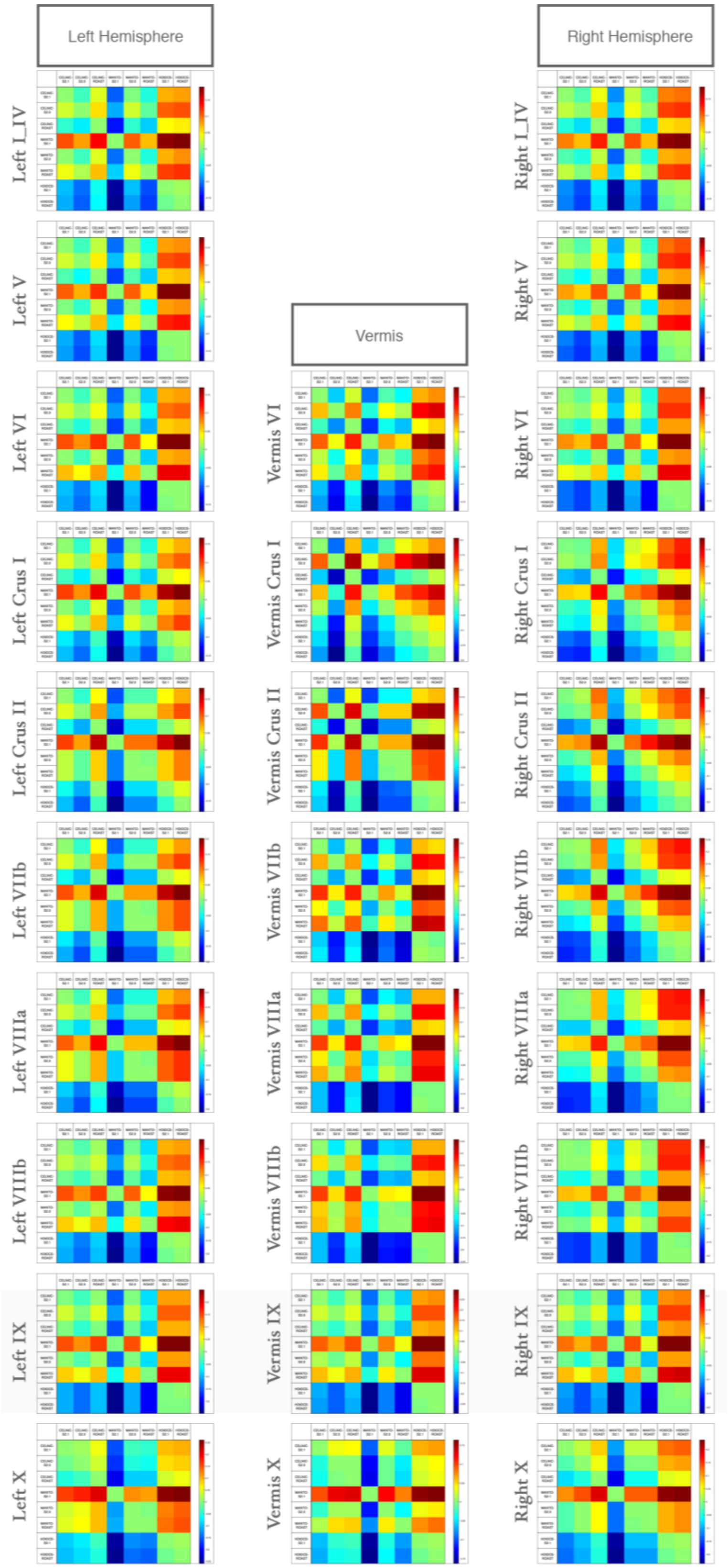

Figure 3. Differences in the lobular electric field strength to different computational pipelines (SimNIBS version 2.1, S2.1; SimNIBS version 2.0, S2.0, and ROAST) for Celnik, Manto, and 4x1 HD-ctDCS montages.

Supplementary Figure 3

Supplementary Figure 4

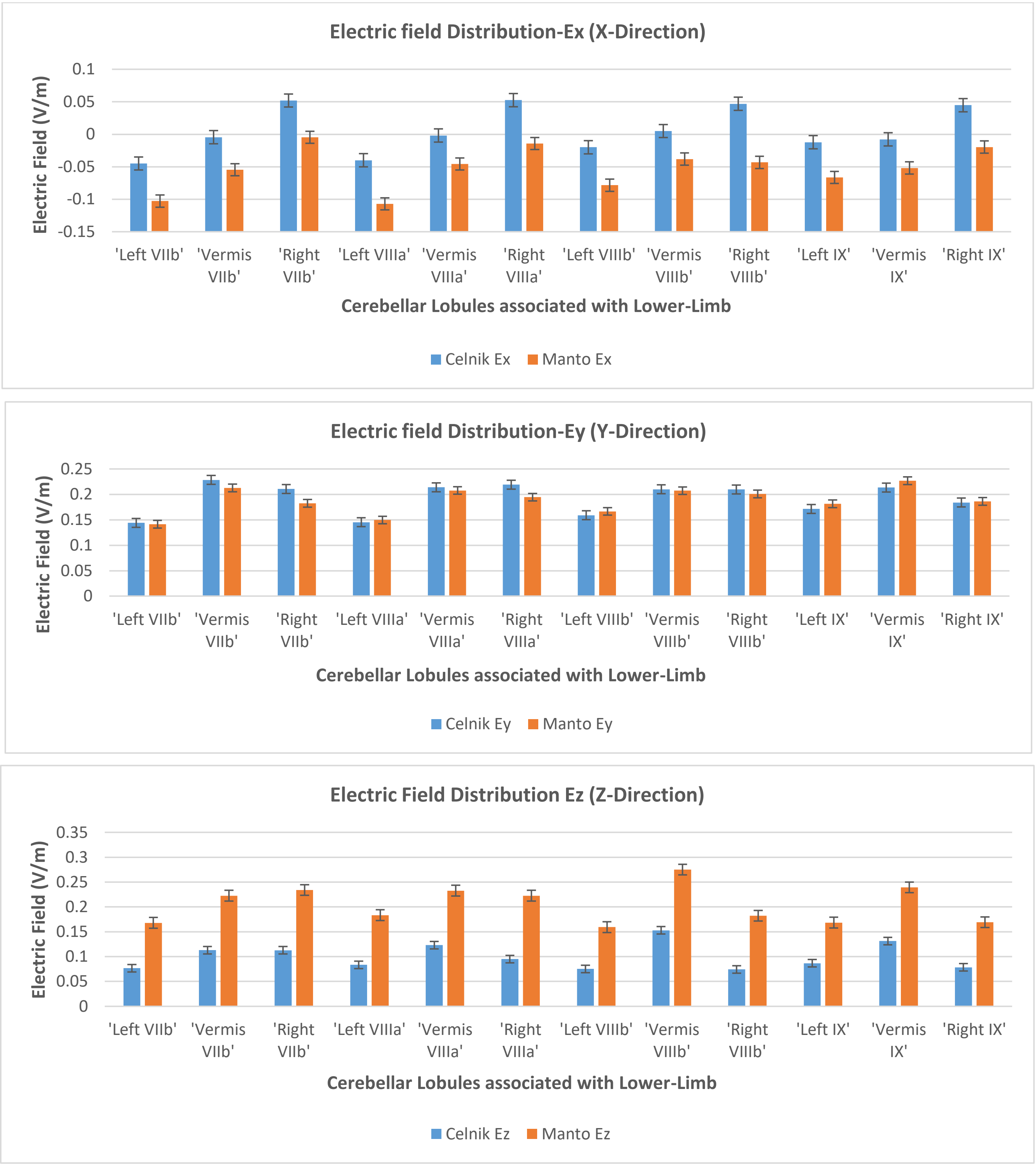

Figure 4. Electric Field Distribution in X, Y, Z directions for the lobules associated with lower-limb function. In E_x direction, Celnik montage performs better than Manto while in E_z direction, Manto performs better than Celnik montage.

Supplementary Figure 5

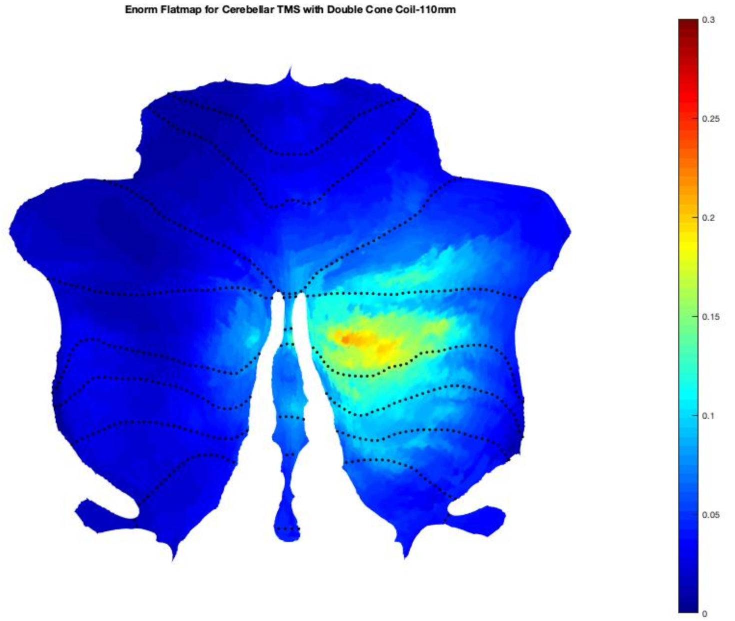

Figure 5. Electric Field (E_{norm}) Distribution for the double-cone coil (110mm-diameter, Magstim, UK) placed at the right cerebellar cortex (3cm lateral to the inion) with the current in the coil directed downwards.

Supplementary Table 1. Electric field strength (V/m) mean±standard deviation at cerebellar lobules due to Celnik ctDCS montage (right side is the targeted side – not much spillover to the contralateral left side as shown with the grayed boxes)

Cerebellar Location	Left side (V/m)	Right side (V/m)	Vermis (V/m)
	EF mean±std	EF mean±std	
I-IV	0.2930±0.1269	0.3206±0.1754	
V	0.2763±0.0154	0.3248±0.2002	
VI	0.3022±0.1665	0.4180±0.2160	0.4509±0.0160
Cr I	0.3351±0.1335	0.4698±0.2305	0.3762±0.2382
Cr II	0.3341±0.1162	0.4531±0.1795	0.5461±0.1153
VIIb	0.3291±0.0112	0.4657±0.1671	0.5162±0.0121
VIIIa	0.3275±0.1356	0.4447±0.1975	0.5002±0.0721
VIIIb	0.3405±0.1375	0.4003±0.1939	0.4961±0.0580
IX	0.4068±0.1693	0.4496±0.1379	0.5383±0.0319
X	0.3590±0.1396	0.4982±0.2530	0.2308±0.1894

Supplementary Table 2. Electric field strength (V/m) mean±standard deviation at cerebellar lobules due to Manto ctDCS montage (right side is the targeted side – spillover to the contralateral left side as shown with the grayed boxes)

**Cerebellar Location**	**Left side (V/m)**	**Right side (V/m)**	**Vermis (V/m)**
	**EF mean±std**	**EF mean±std**	
I-IV	0.3518±0.1533	0.3179±0.1690	
V	0.3347±0.1837	0.3047±0.1865	
VI	0.3740±0.2029	0.3669±0.1876	0.4694±0.1644
Cr I	0.4151±0.1661	0.4092±0.1995	0.3929±0.2484
Cr II	0.3910±0.1422	0.4141±0.1561	0.5496±0.1176
VIIb	0.4012±0.1561	0.4130±0.1419	0.5346±0.0104
VIIIa	0.4219±0.1896	0.3772±0.1606	0.5184±0.0740
VIIIb	0.4300±0.1866	0.3451±0.1637	0.5248±0.0638
IX	0.4563±0.1861	0.4484±0.1459	0.5689±0.0351
X	0.5333±0.2084	0.3839±0.1889	0.2522±0.2044

Supplementary Table 3. Electric field strength (V/m) mean±standard deviation at cerebellar lobules due to HD-tDCS montage (right side is the targeted side – not much spillover to the contralateral left side as shown with the grayed boxes)

Cerebellar Location	Left side (V/m)	Right side (V/m)	Vermis (V/m)
	EF mean±std	EF mean±std	
I-IV	0.0558±0.0254	0.0571±0.0328	
V	0.0539±0.0320	0.0632±0.0396	
VI	0.0646±0.0384	0.0903±0.0485	0.1157±0.0423
Cr I	0.0785±0.0328	0.1076±0.0555	0.0934±0.0595
Cr II	0.0787±0.0275	0.1082±0.0392	0.1425±0.0316
VIIb	0.0657±0.0224	0.0929±0.0321	0.1199±0.0062
VIIIa	0.0581±0.0237	0.0849±0.0340	0.1084±0.0182
VIIIb	0.0569±0.0233	0.0690±0.0345	0.1008±0.0119
IX	0.0735±0.0323	0.0819±0.0262	0.0967±0.0055
X	0.0527±0.0208	0.0761±0.0375	0.0382±0.0309

Supplementary Table 4. Optimization results showing the current magnitude (+ is an anode and – is a cathode) at different electrode locations to align the electric field in X, Y, and Z directions at different lobules combinations (Right VIIb-VIII-IX, VIIb-VII-IX, Right CrusII-VIIb-VIII-IX).

**Right VIIb-VIII-IX**	**x**	**y**	**z**
CzAF10h	0	0	0
CzAF1	0	0	0
CzAF1h	0	0	0
CzAF2	0	0	0
CzAF2h	0	0	0
CzAF3	0	0	0
CzAF3h	0	0	0
CzAF4	0	0	0
CzAF4h	0	0	0
CzAF5	0	0	0
CzAF5h	0	0	0
CzAF6	0	0	0
CzAF6h	0	0	0
CzAF7	0	0	0
CzAF7h	0	0	0
CzAF8	0	0	0
CzAF8h	0	0	0
CzAF9h	0	0	0
CzAFF10	0	0	0
CzAFF10h	0	0	0
CzAFF1	0	0	0
CzAFF1h	0	0	0
CzAFF2	0	0	0
CzAFF2h	0	0	0
CzAFF3	0	0	0
CzAFF3h	0	0	0
CzAFF4	0	0	0
CzAFF4h	0	0	0
CzAFF5	0	0	0
CzAFF5h	0	0	0
CzAFF6	0	0	0
CzAFF6h	0	0	0
CzAFF7	0	0	0
CzAFF7h	0	0	0
CzAFF8	0	0	0
CzAFF8h	0	0	0
CzAFF9	0	0	0

CzAFF9h	0	0	0
CzAFFz	0	0	0
CzAFp10h	0	0	0
CzAFp1	0	0	0
CzAFp1h	0	0	0
CzAFp2	0	0	0
CzAFp2h	0	0	0
CzAFp3	0	0	0
CzAFp3h	0	0	0
CzAFp4	0	0	0
CzAFp4h	0	0	0
CzAFp5	0	0	0
CzAFp5h	0	0	0
CzAFp6	0	0	0
CzAFp6h	0	0	0
CzAFp7	0	0	0
CzAFp7h	0	0	0
CzAFp8	0	0	0
CzAFp8h	0	0	0
CzAFp9h	0	0	0
CzAFpz	0	0	0
CzAFz	0	0	0
CzC1	0	0	0
CzC1h	0	0	0
CzC2	0	0	0
CzC2h	0	0	0
CzC3	0	0	0
CzC3h	0	0	0
CzC4	0	0	0
CzC4h	0	0	0
CzC5	0	0	0
CzC5h	0	0	0
CzC6	0	0	0
CzC6h	0	0	0
CzCCP1	0	0	0
CzCCP1h	0	0	0
CzCCP2	0	0	0
CzCCP2h	0	0	0
CzCCP3	0	0	0
CzCCP3h	0	0	0
CzCCP4	0	0	0
CzCCP4h	0	0	0

CzCCP5	0	0	0
CzCCP5h	0	0	0
CzCCP6	0	0	0
CzCCP6h	0	0	0
CzCCPz	0	0	0
CzCP1	0	0	0
CzCP1h	0	0	0
CzCP2	0	0	0
CzCP2h	0	0	0
CzCP3	0	0	0
CzCP3h	0	0	0
CzCP4	0	0	0
CzCP4h	0	0	0
CzCP5	0	0	0
CzCP5h	0	0	0
CzCP6	0	0	0
CzCP6h	0	0	0
CzCPP1	0	0	0
CzCPP1h	0	0	0
CzCPP2	0	0	0
CzCPP2h	0	0	0
CzCPP3	0	0	0
CzCPP3h	0	0	0
CzCPP4	0	0	0
CzCPP4h	0	0	0
CzCPP5	0	0	0
CzCPP5h	0	0	0
CzCPP6	0	0	0
CzCPP6h	0	0	0
CzCPPz	0	0	0
CzCPz	0	0	0
CzE145	0	-0.19287741	0.088684917
CzE165	0	0	0
CzE216	0	-0.999999976	0
CzE229	0	-0.326783337	0
CzE233	0	0	0
CzE236	0	0	0
CzE237	0	0	0
CzE238	0	0	0
CzE239	0	0	0
CzE240	0	0	0
CzE241	0	0	0

CzE242	0	0	0
CzE243	0	0	0
CzE246	0	0	0
CzE247	0	0	0
CzE251	0	0	0
CzE256	0	0	0
CzE91	0	0	0
CzEx10	0	0	0
CzEx11	0	0	0
CzEx12	0	0	0
CzEx13	0	0	0
CzEx14	0	0	0
CzEx19	0	0	0
CzEx1	0	0	0
CzEx20	0	0	0
CzEx21	0	0	0
CzEx22	0	0	0
CzEx23	0	0	0
CzEx24	0	0	0
CzEx25	0	0	0
CzEx26	0	0	0
CzEx27	0	0	0
CzEx28	0	0	0
CzEx29	0	0	0
CzEx2	0	0	0
CzEx30	0	0	0
CzEx31	0	0	0
CzEx3	0	0	-0.647492514
CzEx4	0	0.999999995	0
CzEx5	0	0	0
CzEx6	0	0	0.911314734
CzEx7	0	0	0
CzEx8	-0.999999987	0	0
CzEx9	0	0	0
CzExx10	0	0	0
CzExx11	0	0	0
CzExx12	0	0	0
CzExx13	0	0	0
CzExx14	0	-0.999997014	0
CzExx19	0	0	0
CzExx1	0	0	0
CzExx20	0	0	0

CzExx21	0	0	0
CzExx22	0	0	0
CzExx23	0	0	0
CzExx24	0	0	0
CzExx25	0	0	0
CzExx26	0	0	0
CzExx27	0	0	0
CzExx28	0	0	0
CzExx29	0	0	0
CzExx2	0	0	0
CzExx30	0	0	0
CzExx31	0	0	0
CzExx32	0	0	0
CzExx33	0	0	0
CzExx34	0	0	0
CzExx3	0	0	0
CzExx4	0	0	0.999999997
CzExx5	0	0	0
CzExx6	0	0	0.999999997
CzExx7	0	0	0
CzExx8	0	0	0.999999996
CzExx9	0	0	0
CzExxz	0	0	0
CzExz	0	0	0
CzF10	0	0	0
CzF10h	0	0	0
CzF1	0	0	0
CzF1h	0	0	0
CzF2	0	0	0
CzF2h	0	0	0
CzF3	0	0	0
CzF3h	0	0	0
CzF4	0	0	0
CzF4h	0	0	0
CzF5	0	0	0
CzF5h	0	0	0
CzF6	0	0	0
CzF6h	0	0	0
CzF7	0	0	0
CzF7h	0	0	0
CzF8	0	0	0
CzF8h	0	0	0

CzF9	0	0	0
CzF9h	0	0	0
CzFC1	0	0	0
CzFC1h	0	0	0
CzFC2	0	0	0
CzFC2h	0	0	0
CzFC3	0	0	0
CzFC3h	0	0	0
CzFC4	0	0	0
CzFC4h	0	0	0
CzFC5	0	0	0
CzFC5h	0	0	0
CzFC6	0	0	0
CzFC6h	0	0	0
CzFCC1	0	0	0
CzFCC1h	0	0	0
CzFCC2	0	0	0
CzFCC2h	0	0	0
CzFCC3	0	0	0
CzFCC3h	0	0	0
CzFCC4	0	0	0
CzFCC4h	0	0	0
CzFCC5	0	0	0
CzFCC5h	0	0	0
CzFCC6	0	0	0
CzFCC6h	0	0	0
CzFCCz	0	0	0
CzFCz	0	0	0
CzFFC1	0	0	0
CzFFC1h	0	0	0
CzFFC2	0	0	0
CzFFC2h	0	0	0
CzFFC3	0	0	0
CzFFC3h	0	0	0
CzFFC4	0	0	0
CzFFC4h	0	0	0
CzFFC5	0	0	0
CzFFC5h	0	0	0
CzFFC6	0	0	0
CzFFC6h	0	0	0
CzFFCz	0	0	0
CzFFT10	0	0	0

CzFFT10h	0	0	0
CzFFT7	0	0	0
CzFFT7h	0	0	0
CzFFT8	0	0	0
CzFFT8h	0	0	0
CzFFT9	0	0	0
CzFFT9h	0	0	0
CzFT10	0	0	0
CzFT10h	0	0	0
CzFT7	0	0	0
CzFT7h	0	0	0
CzFT8	0	0	0
CzFT8h	0	0	0
CzFT9	0	0	0
CzFT9h	0	0	0
CzFTT10	0	0	0
CzFTT10h	0	0	0
CzFTT7	0	0	0
CzFTT7h	0	0	0
CzFTT8	0	0	0
CzFTT8h	0	0	0
CzFTT9	0	0	0
CzFTT9h	0	0	0
CzFp1	0	0	0
CzFp1h	0	0	0
CzFp2	0	0	0
CzFp2h	0	0	0
CzFpz	0	0	0
CzFz	0	0	0
CzI1	0	0	0
CzI1h	0	0	0
CzI2	0	0	0
CzI2h	0	0	0
CzIz	0	0	0
CzO10	0	0.999999892	0
CzO1	0	0	0
CzO1h	0	0	0
CzO2	0	0	0
CzO2h	0	0	0
CzO9	0	0	0
CzOI1	0	0	0
CzOI1h	0	0	0

CzOI2	0	0	0
CzOI2h	0	0	0
CzOIz	0	0	0
CzOz	0	0	0
CzP10	-0.27934424	0	0
CzP10h	0	0	-0.999999974
CzP1	0	0	0
CzP1h	0	0	0
CzP2	0	0	0
CzP2h	0	0	0
CzP3	0	0	0
CzP3h	0	0	0
CzP4	0	0	0
CzP4h	0	0	0
CzP5	0	0	0
CzP5h	0	0	0
CzP6	0	0	0
CzP6h	0	0	0
CzP7	0	0	0
CzP7h	0	0	0
CzP8	0	0	0
CzP8h	0	0	0
CzP9	0	0	0
CzP9h	0	0	0
CzPO10	0	0	0
CzPO10h	-0.999999989	0	0
CzPO1	0	0	0
CzPO1h	0	0	0
CzPO2	0	0	0
CzPO2h	0	0	0
CzPO3	0	0	0
CzPO3h	0	0	0
CzPO4	0	0	0
CzPO4h	0	0	0
CzPO5	0	0	0
CzPO5h	0	0	0
CzPO6	0	0	0
CzPO6h	0	0	0
CzPO7	0	0	0
CzPO7h	0	0	0
CzPO8	0	0	0
CzPO8h	0	0	0

CzPO9	0	0	0
CzPO9h	0	0	0
CzPOO10	0	0.99999999	0
CzPOO10h	0	0	-0.404490138
CzPOO1	0	0	0
CzPOO1h	0	0	0
CzPOO2	0	0	0
CzPOO2h	0	0	0
CzPOO3	0	0	0
CzPOO3h	0	0	0
CzPOO4	0	0	0
CzPOO4h	0	0	0
CzPOO5	0	-0.240172536	0
CzPOO5h	0	0	0
CzPOO6	0	0	0
CzPOO6h	0	0	0
CzPOO7	0	-0.240168757	0
CzPOO7h	0	-0.999999801	0
CzPOO8	0	0	0
CzPOO8h	0	0	0
CzPOO9	0	0	0
CzPOO9h	0	0	0
CzPOOz	0	0	0
CzPOz	0	0	0
CzPPO10	-0.999999985	0	0
CzPPO10h	0	0	-0.99999998
CzPPO1	0	0	0
CzPPO1h	0	0	0
CzPPO2	0	0	0
CzPPO2h	0	0	0
CzPPO3	0	0	0
CzPPO3h	0	0	0
CzPPO4	0	0	0
CzPPO4h	0	0	0
CzPPO5	0	0	0
CzPPO5h	0	0	0
CzPPO6	0	0	0
CzPPO6h	0	0	0
CzPPO7	0	0	0
CzPPO7h	-0.720655161	0	0
CzPPO8	0	0	0
CzPPO8h	0	0	0

CzPPO9	0	0	0
CzPPO9h	0	0	0
CzPPOz	0	0	0
CzPz	0	0	0
CzT10	0	0	0
CzT10h	0	0	0
CzT3	0	0	0
CzT4	0	0	0
CzT5	0	0	0
CzT6	0	0	0
CzT7	0	0	0
CzT7h	0	0	0
CzT8	0	0	0
CzT8h	0	0	0
CzT9	0	0	0
CzT9h	0	0	0
CzTP10	0	0	0
CzTP7	0	0	0
CzTP7h	0	0	0
CzTP8	0	0	0
CzTP8h	0	0	0
CzTP9	0	0	0
CzTPP10h	0	0	0
CzTPP7	0	0	0
CzTPP7h	0	0	0
CzTPP8	0	0	0
CzTPP8h	0	0	0
CzTPP9	0	0	0
CzTPP9h	0	0	0
CzTTP7	0	0	0
CzTTP7h	0	0	0
CzTTP8	0	0	0
CzTTP8h	0	0	0
CzZ10	0	0	0
CzZ11	0	0	0
CzZ1	0.999999983	0	0
CzZ2	0	0	0
CzZ3	0.999999982	0	0
CzZ4	0	0	0
CzZ5	0.999999958	0	0
CzZ6	0	0	0
CzZ7	0	0	-0.948016885

CzZ8	0	0.999999987	0
CzZ9	0.999999863	0	0

**VIIb-VII-IX**	**x**	**y**	**z**
CzAF10h	0	0	0
CzAF1	0	0	0
CzAF1h	0	0	0
CzAF2	0	0	0
CzAF2h	0	0	0
CzAF3	0	0	0
CzAF3h	0	0	0
CzAF4	0	0	0
CzAF4h	0	0	0
CzAF5	0	0	0
CzAF5h	0	0	0
CzAF6	0	0	0
CzAF6h	0	0	0
CzAF7	0	0	0
CzAF7h	0	0	0
CzAF8	0	0	0
CzAF8h	0	0	0
CzAF9h	0	0	0
CzAFF10	0	0	0
CzAFF10h	0	0	0
CzAFF1	0	0	0
CzAFF1h	0	0	0
CzAFF2	0	0	0
CzAFF2h	0	0	0
CzAFF3	0	0	0
CzAFF3h	0	0	0
CzAFF4	0	0	0
CzAFF4h	0	0	0
CzAFF5	0	0	0
CzAFF5h	0	0	0
CzAFF6	0	0	0
CzAFF6h	0	0	0
CzAFF7	0	0	0
CzAFF7h	0	0	0
CzAFF8	0	0	0
CzAFF8h	0	0	0

CzAFF9	0	0	0
CzAFF9h	0	0	0
CzAFFz	0	0	0
CzAFp10h	0	0	0
CzAFp1	0	0	0
CzAFp1h	0	0	0
CzAFp2	0	0	0
CzAFp2h	0	0	0
CzAFp3	0	0	0
CzAFp3h	0	0	0
CzAFp4	0	0	0
CzAFp4h	0	0	0
CzAFp5	0	0	0
CzAFp5h	0	0	0
CzAFp6	0	0	0
CzAFp6h	0	0	0
CzAFp7	0	0	0
CzAFp7h	0	0	0
CzAFp8	0	0	0
CzAFp8h	0	0	0
CzAFp9h	0	0	0
CzAFpz	0	0	0
CzAFz	0	0	0
CzC1	0	0	0
CzC1h	0	0	0
CzC2	0	0	0
CzC2h	0	0	0
CzC3	0	0	0
CzC3h	0	0	0
CzC4	0	0	0
CzC4h	0	0	0
CzC5	0	0	0
CzC5h	0	0	0
CzC6	0	0	0
CzC6h	0	0	0
CzCCP1	0	0	0
CzCCP1h	0	0	0
CzCCP2	0	0	0
CzCCP2h	0	0	0
CzCCP3	0	0	0
CzCCP3h	0	0	0
CzCCP4	0	0	0

CzCCP4h	0	0	0
CzCCP5	0	0	0
CzCCP5h	0	0	0
CzCCP6	0	0	0
CzCCP6h	0	0	0
CzCCPz	0	0	0
CzCP1	0	0	0
CzCP1h	0	0	0
CzCP2	0	0	0
CzCP2h	0	0	0
CzCP3	0	0	0
CzCP3h	0	0	0
CzCP4	0	0	0
CzCP4h	0	0	0
CzCP5	0	0	0
CzCP5h	0	0	0
CzCP6	0	0	0
CzCP6h	0	0	0
CzCPP1	0	0	0
CzCPP1h	0	0	0
CzCPP2	0	0	0
CzCPP2h	0	0	0
CzCPP3	0	0	0
CzCPP3h	0	0	0
CzCPP4	0	0	0
CzCPP4h	0	0	0
CzCPP5	0	0	0
CzCPP5h	0	0	0
CzCPP6	0	0	0
CzCPP6h	0	0	0
CzCPPz	0	0	0
CzCPz	0	0	0
CzE145	0	-0.999999998	-0.542529121
CzE165	0	0	0
CzE216	0	0	0
CzE229	0	0	0
CzE233	0	0	0
CzE236	0	0	0
CzE237	0	-0.999998771	0
CzE238	0	0	0
CzE239	0	0	0
CzE240	0	0	0

CzE241	0	0	0
CzE242	0	0	0
CzE243	0	0	0
CzE246	0	0	0
CzE247	0	0	0
CzE251	0	0	0
CzE256	0	0	0
CzE91	0	0	0
CzEx10	0	0	0
CzEx11	0	0	0
CzEx12	0	0	0
CzEx13	0	0	0
CzEx14	0	0	0
CzEx19	0	0	0
CzEx1	0	0	0
CzEx20	0	0	0
CzEx21	0	0	0
CzEx22	0	0	0
CzEx23	0	0	0
CzEx24	0	0	0
CzEx25	0	0	0
CzEx26	0	0	0
CzEx27	0	0	0
CzEx28	0	0	0
CzEx29	0	0	0
CzEx2	0	0	0
CzEx30	0	0	0
CzEx31	0	0	0
CzEx3	0.857758599	0	0
CzEx4	0	0	0
CzEx5	0.999999994	0	0
CzEx6	-0.999999929	0	0
CzEx7	0.999999994	0	0
CzEx8	-0.999999967	0	0
CzEx9	0.999999981	0	0
CzExx10	0	0	0
CzExx11	0	0	0
CzExx12	0	0	0
CzExx13	0	0	0
CzExx14	0	0	0
CzExx19	0	0	0
CzExx1	0	0	0

CzExx20	0	0	0
CzExx21	0	0	0
CzExx22	0	0	0
CzExx23	0	0	0
CzExx24	0	0	0
CzExx25	0	0	0
CzExx26	0	0	0
CzExx27	0	0	0
CzExx28	0	0	0
CzExx29	0	0	0
CzExx2	0	0	0
CzExx30	0	0	0
CzExx31	0	0	0
CzExx32	0	0	0
CzExx33	0	0	0
CzExx34	0	0	0
CzExx3	0	0	0
CzExx4	0	0	0
CzExx5	0	0	0
CzExx6	0	0	0
CzExx7	0	0	0
CzExx8	0	0	0
CzExx9	0	0	0
CzExxz	0	0	0
CzExz	0	0	0
CzF10	0	0	0
CzF10h	0	0	0
CzF1	0	0	0
CzF1h	0	0	0
CzF2	0	0	0
CzF2h	0	0	0
CzF3	0	0	0
CzF3h	0	0	0
CzF4	0	0	0
CzF4h	0	0	0
CzF5	0	0	0
CzF5h	0	0	0
CzF6	0	0	0
CzF6h	0	0	0
CzF7	0	0	0
CzF7h	0	0	0
CzF8	0	0	0

CzF8h	0	0	0
CzF9	0	0	0
CzF9h	0	0	0
CzFC1	0	0	0
CzFC1h	0	0	0
CzFC2	0	0	0
CzFC2h	0	0	0
CzFC3	0	0	0
CzFC3h	0	0	0
CzFC4	0	0	0
CzFC4h	0	0	0
CzFC5	0	0	0
CzFC5h	0	0	0
CzFC6	0	0	0
CzFC6h	0	0	0
CzFCC1	0	0	0
CzFCC1h	0	0	0
CzFCC2	0	0	0
CzFCC2h	0	0	0
CzFCC3	0	0	0
CzFCC3h	0	0	0
CzFCC4	0	0	0
CzFCC4h	0	0	0
CzFCC5	0	0	0
CzFCC5h	0	0	0
CzFCC6	0	0	0
CzFCC6h	0	0	0
CzFCCz	0	0	0
CzFCz	0	0	0
CzFFC1	0	0	0
CzFFC1h	0	0	0
CzFFC2	0	0	0
CzFFC2h	0	0	0
CzFFC3	0	0	0
CzFFC3h	0	0	0
CzFFC4	0	0	0
CzFFC4h	0	0	0
CzFFC5	0	0	0
CzFFC5h	0	0	0
CzFFC6	0	0	0
CzFFC6h	0	0	0
CzFFCz	0	0	0

CzFFT10	0	0	0
CzFFT10h	0	0	0
CzFFT7	0	0	0
CzFFT7h	0	0	0
CzFFT8	0	0	0
CzFFT8h	0	0	0
CzFFT9	0	0	0
CzFFT9h	0	0	0
CzFT10	0	0	0
CzFT10h	0	0	0
CzFT7	0	0	0
CzFT7h	0	0	0
CzFT8	0	0	0
CzFT8h	0	0	0
CzFT9	0	0	0
CzFT9h	0	0	0
CzFTT10	0	0	0
CzFTT10h	0	0	0
CzFTT7	0	0	0
CzFTT7h	0	0	0
CzFTT8	0	0	0
CzFTT8h	0	0	0
CzFTT9	0	0	0
CzFTT9h	0	0	0
CzFp1	0	0	0
CzFp1h	0	0	0
CzFp2	0	0	0
CzFp2h	0	0	0
CzFpz	0	0	0
CzFz	0	0	0
CzI1	0	0	0
CzI1h	0	0.999999971	0
CzI2	0	0	0
CzI2h	0	0.999999969	0
CzIz	0	0.806213136	0
CzO10	0	0	0
CzO1	0	0	0
CzO1h	0	0	0
CzO2	0	0	0
CzO2h	0	0	0
CzO9	0	0.999999949	0
CzOI1	0	0	0

CzOI1h	0	0	0
CzOI2	0	0	0
CzOI2h	0	0	0
CzOIz	0	0	0
CzOz	0	0	0
CzP10	0	0	0
CzP10h	0	0	0
CzP1	0	0	0
CzP1h	0	0	0
CzP2	0	0	0
CzP2h	0	0	0
CzP3	0	0	0
CzP3h	0	0	0
CzP4	0	0	0
CzP4h	0	0	0
CzP5	0	0	0
CzP5h	0	0	0
CzP6	0	0	0
CzP6h	0	0	0
CzP7	0	0	0
CzP7h	0	0	0
CzP8	0	0	-0.999999389
CzP8h	0	0	0
CzP9	0	0	0
CzP9h	0	0	0
CzPO10	0	0	0
CzPO10h	-0.999999966	0	0
CzPO1	0	0	0
CzPO1h	0	0	0
CzPO2	0	0	0
CzPO2h	0	0	0
CzPO3	0	0	0
CzPO3h	0	0	0
CzPO4	0	0	0
CzPO4h	0	0	0
CzPO5	0	0	0
CzPO5h	0	0	0
CzPO6	0	0	0
CzPO6h	0	0	0
CzPO7	0	0	-0.999994767
CzPO7h	0	0	0
CzPO8	0	0	0

CzPO8h	0	0	0
CzPO9	0	0	0
CzPO9h	0	0	-0.999999914
CzPOO10	-0.999999711	0	0
CzPOO10h	0	0	0
CzPOO1	0	0	0
CzPOO1h	0	0	0
CzPOO2	0	0	0
CzPOO2h	0	0	0
CzPOO3	0	0	0
CzPOO3h	0	0	0
CzPOO4	0	0	0
CzPOO4h	0	0	0
CzPOO5	0	0	0
CzPOO5h	0	0	0
CzPOO6	0	0	0
CzPOO6h	0	0	0
CzPOO7	0	0	0
CzPOO7h	0	0	0
CzPOO8	0	0	-0.158135922
CzPOO8h	0	0	0
CzPOO9	0.142240344	0	0
CzPOO9h	0	0	0
CzPOOz	0	0	0
CzPOz	0	0	0
CzPPO10	0	0	0
CzPPO10h	0	0	0
CzPPO1	0	0	0
CzPPO1h	0	0	0
CzPPO2	0	0	0
CzPPO2h	0	0	0
CzPPO3	0	0	0
CzPPO3h	0	0	0
CzPPO4	0	0	0
CzPPO4h	0	0	0
CzPPO5	0	0	0
CzPPO5h	0	0	0
CzPPO6	0	0	0
CzPPO6h	0	0	0
CzPPO7	0	0	0
CzPPO7h	0	0	0
CzPPO8	0	0	0

CzPPO8h	0	0	0
CzPPO9	0	0	0
CzPPO9h	0	0	-0.299335261
CzPPOz	0	0	0
CzPz	0	0	0
CzT10	0	0	0
CzT10h	0	0	0
CzT3	0	0	0
CzT4	0	0	0
CzT5	0	0	0
CzT6	0	0	0
CzT7	0	0	0
CzT7h	0	0	0
CzT8	0	0	0
CzT8h	0	0	0
CzT9	0	0	0
CzT9h	0	0	0
CzTP10	0	0	0
CzTP7	0	0	0
CzTP7h	0	0	0
CzTP8	0	0	0
CzTP8h	0	0	0
CzTP9	0	0	0
CzTPP10h	0	0	0
CzTPP7	0	0	0
CzTPP7h	0	0	0
CzTPP8	0	0	0
CzTPP8h	0	0	0
CzTPP9	0	0	0
CzTPP9h	0	0	0
CzTTP7	0	0	0
CzTTP7h	0	0	0
CzTTP8	0	0	0
CzTTP8h	0	0	0
CzZ10	0	-0.999999859	0
CzZ11	0	0	0
CzZ1	0	0	0.999999968
CzZ2	0	0	0.999999386
CzZ3	0	0	0.999999987
CzZ4	0	-0.999999962	0
CzZ5	0	0	0
CzZ6	0	0	0

CzZ7	0	0	0
CzZ8	0	0.193786392	0
CzZ9	0	0	0.999999984

**Right CrusII-VIIb-VIII-IX**	**x**	**y**	**z**
CzAF10h	0	0	0
CzAF1	0	0	0
CzAF1h	0	0	0
CzAF2	0	0	0
CzAF2h	0	0	0
CzAF3	0	0	0
CzAF3h	0	0	0
CzAF4	0	0	0
CzAF4h	0	0	0
CzAF5	0	0	0
CzAF5h	0	0	0
CzAF6	0	0	0
CzAF6h	0	0	0
CzAF7	0	0	0
CzAF7h	0	0	0
CzAF8	0	0	0
CzAF8h	0	0	0
CzAF9h	0	0	0
CzAFF10	0	0	0
CzAFF10h	0	0	0
CzAFF1	0	0	0
CzAFF1h	0	0	0
CzAFF2	0	0	0
CzAFF2h	0	0	0
CzAFF3	0	0	0
CzAFF3h	0	0	0
CzAFF4	0	0	0
CzAFF4h	0	0	0
CzAFF5	0	0	0
CzAFF5h	0	0	0
CzAFF6	0	0	0
CzAFF6h	0	0	0
CzAFF7	0	0	0
CzAFF7h	0	0	0
CzAFF8	0	0	0

CzAFF8h	0	0	0
CzAFF9	0	0	0
CzAFF9h	0	0	0
CzAFFz	0	0	0
CzAFp10h	0	0	0
CzAFp1	0	0	0
CzAFp1h	0	0	0
CzAFp2	0	0	0
CzAFp2h	0	0	0
CzAFp3	0	0	0
CzAFp3h	0	0	0
CzAFp4	0	0	0
CzAFp4h	0	0	0
CzAFp5	0	0	0
CzAFp5h	0	0	0
CzAFp6	0	0	0
CzAFp6h	0	0	0
CzAFp7	0	0	0
CzAFp7h	0	0	0
CzAFp8	0	0	0
CzAFp8h	0	0	0
CzAFp9h	0	0	0
CzAFpz	0	0	0
CzAFz	0	0	0
CzC1	0	0	0
CzC1h	0	0	0
CzC2	0	0	0
CzC2h	0	0	0
CzC3	0	0	0
CzC3h	0	0	0
CzC4	0	0	0
CzC4h	0	0	0
CzC5	0	0	0
CzC5h	0	0	0
CzC6	0	0	0
CzC6h	0	0	0
CzCCP1	0	0	0
CzCCP1h	0	0	0
CzCCP2	0	0	0
CzCCP2h	0	0	0
CzCCP3	0	0	0
CzCCP3h	0	0	0

CzCCP4	0	0	0
CzCCP4h	0	0	0
CzCCP5	0	0	0
CzCCP5h	0	0	0
CzCCP6	0	0	0
CzCCP6h	0	0	0
CzCCPz	0	0	0
CzCP1	0	0	0
CzCP1h	0	0	0
CzCP2	0	0	0
CzCP2h	0	0	0
CzCP3	0	0	0
CzCP3h	0	0	0
CzCP4	0	0	0
CzCP4h	0	0	0
CzCP5	0	0	0
CzCP5h	0	0	0
CzCP6	0	0	0
CzCP6h	0	0	0
CzCPP1	0	0	0
CzCPP1h	0	0	0
CzCPP2	0	0	0
CzCPP2h	0	0	0
CzCPP3	0	0	0
CzCPP3h	0	0	0
CzCPP4	0	0	0
CzCPP4h	0	0	0
CzCPP5	0	0	0
CzCPP5h	0	0	0
CzCPP6	0	0	0
CzCPP6h	0	0	0
CzCPPz	0	0	0
CzCPz	0	0	0
CzE145	0	0	0
CzE165	0	0	0
CzE216	0	-0.79792297	0
CzE229	0	0	0
CzE233	0	0	0
CzE236	0	0	0
CzE237	0	0	0
CzE238	0	0	0
CzE239	0	0	0

CzE240	0	0	0
CzE241	0	0	0
CzE242	0	0	0
CzE243	0	0	0
CzE246	0	0	0
CzE247	0	0	0
CzE251	0	0	0
CzE256	0	0	0
CzE91	0	0	0
CzEx10	0	0	0
CzEx11	0	0	0
CzEx12	0	0	0
CzEx13	0	0	0
CzEx14	0	-0.999999804	0
CzEx19	0	0	0
CzEx1	0	0	0
CzEx20	0	0	0
CzEx21	0	0	0
CzEx22	0	0	0
CzEx23	0	0	0
CzEx24	0	0	0
CzEx25	0	0	0
CzEx26	0	0	0
CzEx27	0	0	0
CzEx28	0	0	0
CzEx29	0	0	0
CzEx2	0	0	0
CzEx30	0	0	0
CzEx31	0	0	0
CzEx3	0	0	-0.71397973
CzEx4	0	0	0.999999371
CzEx5	0	0	-0.999998561
CzEx6	0	0	0.999999988
CzEx7	0	0	0
CzEx8	0	0	0
CzEx9	0	0	0
CzExx10	0	0	0
CzExx11	0	0	0
CzExx12	0	0	0
CzExx13	0	0	0
CzExx14	0	-0.99999996	0
CzExx19	0	0	0

CzExx1	0	0	0
CzExx20	0	0	0
CzExx21	0	0	0
CzExx22	0	0	0
CzExx23	0	0	0
CzExx24	0	0	0
CzExx25	0	0	0
CzExx26	0	0	0
CzExx27	0	0	0
CzExx28	0	0	0
CzExx29	0	0	0
CzExx2	0	0	0
CzExx30	0	0	0
CzExx31	0	0	0
CzExx32	0	0	0
CzExx33	0	0	0
CzExx34	0	0	0
CzExx3	0	0	0
CzExx4	0	0	0.999999988
CzExx5	0	0	-0.324410911
CzExx6	0	0	0.999999981
CzExx7	0	0	0
CzExx8	0	0	0
CzExx9	0	0	0
CzExxz	0	0	0
CzExz	0	0	0
CzF10	0	0	0
CzF10h	0	0	0
CzF1	0	0	0
CzF1h	0	0	0
CzF2	0	0	0
CzF2h	0	0	0
CzF3	0	0	0
CzF3h	0	0	0
CzF4	0	0	0
CzF4h	0	0	0
CzF5	0	0	0
CzF5h	0	0	0
CzF6	0	0	0
CzF6h	0	0	0
CzF7	0	0	0
CzF7h	0	0	0

CzF8	0	0	0
CzF8h	0	0	0
CzF9	0	0	0
CzF9h	0	0	0
CzFC1	0	0	0
CzFC1h	0	0	0
CzFC2	0	0	0
CzFC2h	0	0	0
CzFC3	0	0	0
CzFC3h	0	0	0
CzFC4	0	0	0
CzFC4h	0	0	0
CzFC5	0	0	0
CzFC5h	0	0	0
CzFC6	0	0	0
CzFC6h	0	0	0
CzFCC1	0	0	0
CzFCC1h	0	0	0
CzFCC2	0	0	0
CzFCC2h	0	0	0
CzFCC3	0	0	0
CzFCC3h	0	0	0
CzFCC4	0	0	0
CzFCC4h	0	0	0
CzFCC5	0	0	0
CzFCC5h	0	0	0
CzFCC6	0	0	0
CzFCC6h	0	0	0
CzFCCz	0	0	0
CzFCz	0	0	0
CzFFC1	0	0	0
CzFFC1h	0	0	0
CzFFC2	0	0	0
CzFFC2h	0	0	0
CzFFC3	0	0	0
CzFFC3h	0	0	0
CzFFC4	0	0	0
CzFFC4h	0	0	0
CzFFC5	0	0	0
CzFFC5h	0	0	0
CzFFC6	0	0	0
CzFFC6h	0	0	0

CzFFCz	0	0	0
CzFFT10	0	0	0
CzFFT10h	0	0	0
CzFFT7	0	0	0
CzFFT7h	0	0	0
CzFFT8	0	0	0
CzFFT8h	0	0	0
CzFFT9	0	0	0
CzFFT9h	0	0	0
CzFT10	0	0	0
CzFT10h	0	0	0
CzFT7	0	0	0
CzFT7h	0	0	0
CzFT8	0	0	0
CzFT8h	0	0	0
CzFT9	0	0	0
CzFT9h	0	0	0
CzFTT10	0	0	0
CzFTT10h	0	0	0
CzFTT7	0	0	0
CzFTT7h	0	0	0
CzFTT8	0	0	0
CzFTT8h	0	0	0
CzFTT9	0	0	0
CzFTT9h	0	0	0
CzFp1	0	0	0
CzFp1h	0	0	0
CzFp2	0	0	0
CzFp2h	0	0	0
CzFpz	0	0	0
CzFz	0	0	0
CzI1	0	0	0
CzI1h	0	0	0
CzI2	0	0	0
CzI2h	0	0	0
CzIz	0	0	0
CzO10	0	0.999999995	0
CzO1	0	0	0
CzO1h	0	0	0
CzO2	0	0	0
CzO2h	0	0	0
CzO9	0	0	0

CzOI1	0	0	0
CzOI1h	0	0	0
CzOI2	0	0	0
CzOI2h	0	0	0
CzOIz	0	0	0
CzOz	0	0	0
CzP10	-0.999999987	0	0
CzP10h	0	0	0
CzP1	0	0	0
CzP1h	0	0	0
CzP2	0	0	0
CzP2h	0	0	0
CzP3	0	0	0
CzP3h	0	0	0
CzP4	0	0	0
CzP4h	0	0	0
CzP5	0	0	0
CzP5h	0	0	0
CzP6	0	0	0
CzP6h	0	0	0
CzP7	0	0	0
CzP7h	0	0	0
CzP8	0	0	-0.96160997
CzP8h	0	0	0
CzP9	0	0	0
CzP9h	0	0	0
CzPO10	0	0.999999893	0
CzPO10h	-0.999999993	0	0
CzPO1	0	0	0
CzPO1h	0	0	0
CzPO2	0	0	0
CzPO2h	0	0	0
CzPO3	0	0	0
CzPO3h	0	0	0
CzPO4	0	0	0
CzPO4h	0	0	0
CzPO5	0	0	0
CzPO5h	0	0	0
CzPO6	0	0	0
CzPO6h	0	0	0
CzPO7	0	0	0
CzPO7h	0	0	0

CzPO8	0	0	0
CzPO8h	0	0	0
CzPO9	0	0	0
CzPO9h	0	0	0
CzPOO10	-0.594240842	0.999999991	0
CzPOO10h	0	0	0
CzPOO1	0	0	0
CzPOO1h	0	0	0
CzPOO2	0	0	0
CzPOO2h	0	0	0
CzPOO3	0	0	0
CzPOO3h	0	0	0
CzPOO4	0	0	0
CzPOO4h	0	0	0
CzPOO5	0	0	0
CzPOO5h	0	0	0
CzPOO6	0	0	0
CzPOO6h	0	0	0
CzPOO7	0	0	0
CzPOO7h	0	-0.641685707	0
CzPOO8	0	0	0
CzPOO8h	0	0	0
CzPOO9	0	0	0
CzPOO9h	0	-0.560390967	0
CzPOOz	0	0	0
CzPOz	0	0	0
CzPPO10	-0.999999984	0	0
CzPPO10h	0	0	0
CzPPO1	0	0	0
CzPPO1h	0	0	0
CzPPO2	0	0	0
CzPPO2h	0	0	0
CzPPO3	0	0	0
CzPPO3h	0	0	0
CzPPO4	0	0	0
CzPPO4h	0	0	0
CzPPO5	0	0	0
CzPPO5h	0	0	0
CzPPO6	0	0	0
CzPPO6h	0	0	0
CzPPO7	-0.287678164	0	0
CzPPO7h	0	0	0

CzPPO8	0	0	-0.999999599
CzPPO8h	0	0	0
CzPPO9	0	0	0
CzPPO9h	0	0	0
CzPPOz	0	0	0
CzPz	0	0	0
CzT10	0	0	0
CzT10h	0	0	0
CzT3	0	0	0
CzT4	0	0	0
CzT5	-0.118080325	0	0
CzT6	0	0	0
CzT7	0	0	0
CzT7h	0	0	0
CzT8	0	0	0
CzT8h	0	0	0
CzT9	0	0	0
CzT9h	0	0	0
CzTP10	0	0	0
CzTP7	0	0	0
CzTP7h	0	0	0
CzTP8	0	0	0
CzTP8h	0	0	0
CzTP9	0	0	0
CzTPP10h	0	0	0
CzTPP7	0	0	0
CzTPP7h	0	0	0
CzTPP8	0	0	0
CzTPP8h	0	0	0
CzTPP9	0	0	0
CzTPP9h	0	0	0
CzTTP7	0	0	0
CzTTP7h	0	0	0
CzTTP8	0	0	0
CzTTP8h	0	0	0
CzZ10	0	0	0
CzZ11	0	0	0
CzZ1	0.999999968	0	0
CzZ2	0	0	0
CzZ3	0.999999983	0	0
CzZ4	0	0	0
CzZ5	0.999997546	0	0

CzZ6	0	0	0
CzZ7	0	0	0
CzZ8	0	0.999999986	0
CzZ9	0.999999978	0	0
